# Distinct mechanisms of the human mitoribosome recycling and antibiotic resistance

**DOI:** 10.1038/s41467-021-23726-4

**Published:** 2021-06-14

**Authors:** Ravi Kiran Koripella, Ayush Deep, Ekansh K. Agrawal, Pooja Keshavan, Nilesh K. Banavali, Rajendra K. Agrawal

**Affiliations:** 1 Division of Translational Medicine, Wadsworth Center, New York State Department of Health, Empire State Plaza, Albany, NY USA; 2 Department of Biomedical Sciences, University at Albany, Albany, NY USA

**Keywords:** Mitochondria, Antibiotics, Ribosome, Cryoelectron microscopy

## Abstract

Ribosomes are recycled for a new round of translation initiation by dissociation of ribosomal subunits, messenger RNA and transfer RNA from their translational post-termination complex. Here we present cryo-EM structures of the human 55S mitochondrial ribosome (mitoribosome) and the mitoribosomal large 39S subunit in complex with mitoribosome recycling factor (RRF_mt_) and a recycling-specific homolog of elongation factor G (EF-G2_mt_). These structures clarify an unusual role of a mitochondria-specific segment of RRF_mt_, identify the structural distinctions that confer functional specificity to EF-G2_mt_, and show that the deacylated tRNA remains with the dissociated 39S subunit, suggesting a distinct sequence of events in mitoribosome recycling. Furthermore, biochemical and structural analyses reveal that the molecular mechanism of antibiotic fusidic acid resistance for EF-G2_mt_ is markedly different from that of mitochondrial elongation factor EF-G1_mt_, suggesting that the two human EF-G_mt_s have evolved diversely to negate the effect of a bacterial antibiotic.

## Abstract (web summary)

High-resolution cryo-EM structures and biochemical analyses of the human mitoribosome, in complex with mitochondria-specific factors mediating mitoribosome recycling, RRFmt and EF-G2mt, offer insight into mechanisms of mitoribosome recycling and resistance to antibiotic fusidic acid.

## Introduction

The process of protein synthesis in all living cells is orchestrated by highly complex macromolecular assemblies called ribosomes, in coordination with mRNA, tRNAs, and multiple translational factors. Mitochondrial ribosomes (mitoribosomes) and their associated translation machinery are distinct from those in the cytoplasm and display features reminiscent of prokaryotic translation^1^, in line with the assumption that mitochondria have evolved from endocytosis of an α-proteobacterium by an ancestral eukaryotic cell^2^. However, cryo-electron microscopy (cryo-EM) structures have revealed that the mammalian mitoribosomes have diverged considerably from bacterial ribosomes and acquired several unique features^3–8^. A striking difference is the reversal in the protein to RNA ratio, as the bacterial ribosomes are high in ribosomal RNA (rRNA) whereas the mammalian mitoribosomes are high in protein. The increase in protein mass is the result of acquisition of multiple mito-specific ribosomal proteins (MRPs) and addition of extensions to many MRPs, that are homologous to bacterial ribosomal proteins. Though the steps of mitochondrial translation closely resemble those for prokaryotic translation in the general sequence of events and the homologous accessory protein factors involved, they also show significant structural and functional differences^9,10^.

The complex process of protein synthesis is accomplished in four essential steps of initiation, elongation, termination, and the ribosome recycling. The transition between translation termination and ribosome recycling is well characterized in eubacteria. During translation termination, the nascent polypeptide chain attached to the peptidyl tRNA is released from the ribosome with the help of a class I release factor (RF) that interacts with the stop codon exposed at the ribosomal decoding site, or aminoacyl-tRNA binding site (A site)^11,12^. Subsequently, the class I RF is dissociated from the ribosome with the help of a class II RF in a GTP hydrolysis-dependent manner^13,14^. At the end of the termination, the translated mRNA and the deacylated tRNA remain associated with the ribosome^15,16^, a state referred to as the post-termination complex (PoTC). In order to initiate a new round of protein synthesis, the ribosome must be split into its two subunits and its bound ligands must be removed. In eubacteria, the disassembly of the PoTC requires the concerted action of two protein factors, the ribosome recycling factor (RRF) and the elongation factor G (EF-G)^15,17,18^. RRF binds to the PoTC as the 70S ribosome adopts a ratcheted conformation^19,20^, in which the small (30S) subunit of the ribosome rotates in an anticlockwise direction with respect to the large (50S) subunit^21^. This is followed by the binding of EF-G in conjugation with guanosine 5′-triphosphate (GTP) to the RRF-bound PoTC and the dissociation of the 70S ribosome into its large and small subunits, a process that requires the hydrolysis of GTP on EF-G^22–24^. Though the involvement of a third factor, initiation factor 3 (IF3) in the recycling process is generally agreed upon, its precise function has been debated^16,18,23^.

Unlike most eubacteria, where a single form of EF-G is known to participate in both the elongation and ribosome recycling steps^22,25^, mammalian mitochondria utilize two isoforms of EF-G, EF-G1_mt_, and EF-G2_mt_^26,27^. While EF-G1_mt_ specifically functions as a translocase during the polypeptide elongation step^28^, EF-G2_mt_ has been reported to act exclusively as a second recycling factor together with RRF_mt_^27^. Human RRF_mt_ is about 25–30% identical to its eubacterial homologs but carries an additional 79 amino acids (aa) long extension at its N-terminus^29^. The recent high-resolution cryo-EM structures of RRF_mt_ bound to the 55S mitoribosomes^7^, and an in vivo formed mitoribosomal complex^30^ revealed that the structurally conserved segment of the RRF_mt_ is similar to its bacterial analog on^14,19,20,31–36^ and off^37,38^ the 70S ribosome in terms of its overall size and domain composition. However, the unique mito-specific N-terminal extension (NTE) in RRF_mt_ extends towards the GTPase-associated center and interacts with the functionally important 16S rRNA elements of the mitoribosomal 39S subunit, including the rRNA helices 89 (H89), H90, and H92^7^.

Valuable mechanistic inferences about the bacterial ribosome recycling process were made from the structures of the 70S•RRF (e.g., Agrawal et al.^31^) and dissociated 50S•RRF•EF-G^14,20^ complexes. Capturing the simultaneous binding of both factors on the 70S ribosome is challenging, however, owing to the rapid rate of 70S ribosomes dissociation into subunits by the combined action of RRF and EF-G^22^. To slow down this reaction, a heterologous system with *T. thermophilus* RRF and *E. coli* EF-G was used to capture both factors on the 70S ribosome by cryo-EM^34^. Subsequently, a time-resolved cryo-EM study was also able to capture various 70S•RRF•EF-G functional intermediates, albeit at low resolution^36^. More recently, a bacterial ribosome recycling complex containing both RRF and EF-G was obtained by X-ray crystallography by stabilizing EF-G on the 70S ribosome through a fusion between EF-G and ribosomal protein bL9^35^. All these structures conclude that binding of EF-G to the 70S•RRF complex induces rotation of RRF domain II towards the helix 44 (h44) region of the 30S subunit, destabilizing the crucial intersubunit bridges B2a and B3, and thereby facilitating the dissociation of the 70S ribosome into its two subunits.

With a molecular weight of 87 kD, human EF-G2_mt_ is slightly larger than EF-G1_mt_ (83 kD), as well as the two isoforms of bacterial EF-Gs, EF-G (78 kD), and EF-G2 (73 kD), identified in certain bacterial species but without known function for the second bacterial isoform^39–41^, except in case of a spirochete^42^. EF-G2_mt_ has about 36% aa sequence identity to EF-G1_mt_ and about 30% aa identity to both its bacterial homologs. Some mammalian mitochondrial translation steps are now better understood through determination of the cryo-EM structures of the initiation^43–45^ and the elongation^6,46^ complexes at high-resolution. Our previous study of the human mitoribosome recycling complex of the RRF_mt_-bound 55S^7^ provided useful insights into the mito-specific aspects of the recycling process, but a complete 55S mitoribosomal recycling complex comprising both RRF_mt_ and EF-G_mt_ remained elusive.

In this work, we investigate the role of mito-specific aa segments of RRF_mt_ and EF-G2_mt_ in recycling the 55S mitoribosome PoTC by determining cryo-EM structures of the key intermediate mitoribosome•RRF_mt_•EF-G2_mt_ complexes, and determine the effect of fusidic acid, an antibiotic that is known to inhibit the GTPase activity of bacterial EF-G^47^, on the GTPase activity of EF-G2_mt_. Our study reveals distinct features of the mechanism of human mitoribosome recycling and of the mechanism of fusidic-acid resistance shown by EF-G1_mt_ and EF-G2_mt_.

## Results and discussion

### Structure of the human mitoribosome recycling complex

**Fig. 1:**
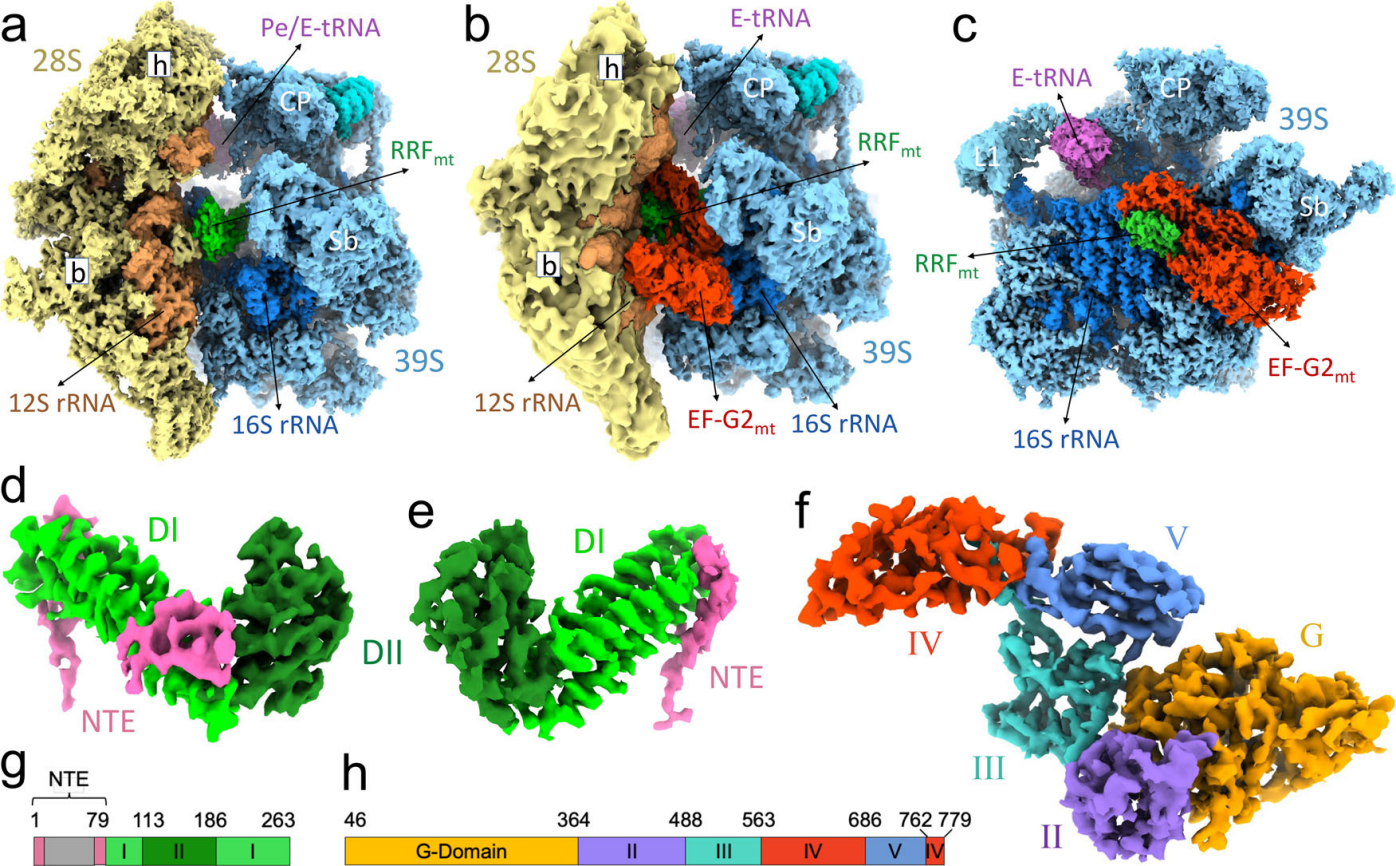
Cryo-EM structures of the human mitochondrial recycling complexes in three functional states. Segmented cryo-EM maps of the mitoribosomal **a** 55S•RRF_mt_ complex (Class I), **b** 55S•RRF_mt_•EF-G2_mt_•GMPPCP complex (Class II), and **c** 39S•RRF_mt_•EF-G2_mt_•GMPPCP complex (Class III). In these three panels, the 28S subunit is shown in yellow, the 39S subunit in blue, E-tRNA in orchid, RRF_mt_ in green and EF-G2_mt_ in red. A lighter shade of yellow differentiates the 28S ribosomal proteins from the 12S rRNA, while a lighter shade of blue differentiates the 39S ribosomal proteins from the 16S rRNA. Landmarks of the 28S subunit: h, head; b, body. Landmarks of the 39S subunit: CP central protuberance, Sb stalk base; L1, MRP uL1m. **d** Cryo-EM density of RRF_mt_ extracted from the Class I complex. **e** Cryo-EM density of RRF_mt_ extracted from the Class III complex. **f** Cryo-EM density of EF-G2_mt_ extracted from Class III complex. See Figs. S1 and S2, for overall and local resolutions, respectively, of densities corresponding to the mitoribosome, RRF_mt_, and EF-G2_mt_ in these complexes. **g** Domain organization in RRF_mt_ displaying domain I (light green), domain II (dark green), modeled region of NTE (pink) and unmodelled region of NTE (gray) **h** Domain organization in EF-G2_mt_ showing G domain (orange), domain II (purple), domain III (cyan), domain IV (red) and domain V (blue).

To investigate the molecular mechanism of ribosome recycling in mammalian mitochondria, we first prepared a model post-termination complex (PoTC) by incubating the human 55S mitoribosome with puromycin^7,48^. The model PoTC was briefly incubated with human RRF_mt_ and human EF-G2_mt_-GMPPCP to obtain the mitoribosome recycling complex. (see “Methods” section). Single-particle cryo-EM analysis on this complex yielded three major classes that each represent a major functional state formed during human mitoribosome recycling, referred henceforth to as Class I, Class II, and Class III (Fig. S1). Class I corresponds to the intact 55S mitoribosome that carries a strong density for RRF_mt,_ and was refined to 3.5 Å (Figs. 1 and S1a). Class II, a relatively small class with only 28,929 particle images, corresponds to the 55S mitoribosome that carries both RRF_mt_ and EF-G2_mt_, and was refined to 3.9 Å (Fig. S2b). In this class, the densities corresponding to the large (39S) mitoribosomal subunit, RRF_mt_, and EF-G2_mt_ are well resolved, but the small (28S) mitoribosomal subunit appears to be loosely bound and present in multiple poses (Figs. 1 and S2). The most populated Class III corresponds to the dissociated 39S subunits that carry both RRF_mt_ and EF-G2_mt_, and was refined to 3.15 Å (Figs. 1 and S2c). Class II likely represents an ensemble of low-population intermediate states of mitoribosome recycling that occur between the states represented by Classes I and III. In addition to these three recycling complexes, we have also obtained a class of particles consisting of 55S mitoribosomes without either of the two factors, where the 28S subunit was rotated by about 8° around its long axis such that its shoulder side moves closer to the 39S subunit while its platform side moves away from it (Fig. S3a). A similar orientation for the small subunit relative to the large subunit, termed as “subunit rolling” has been reported earlier for the 80S ribosomes^49^ and the 55S mitoribosomes^3,7^.

### RRF_mt_ binding stabilizes the rotated state of the 28S subunit in the 55S mitoribosome

Superimposition of our Class I complex with the RRF_mt_-unbound human^3^, bovine^8^ and porcine^4^ 55S mitoribosomes showed that the small 28S subunit was rotated counter-clockwise by about 8.5° with respect to the large 39S subunit (Fig. S3b), similar to the “ratchet-like inter-subunit rotation” observed in the bacterial 70S ribosome^21^ and the 55S mitoribosomes complexed with translational factors^6,7,44,46^. In addition to the inter-subunit rotation, the head domain of the 28S subunit is rotated by about 4° towards the tRNA exit (E) site in a direction roughly orthogonal to the inter-subunit motion (Fig. S3b), similar to “head swiveling” in the bacterial 70S ribosomes^50,51^. As expected, the structure of the Class I complex matches the previously published 3.9 Å resolution map of the analogous 55S•RRF_mt_ complex^7^.

The Class I map showed the characteristic “L” shaped RRF_mt_ density and a density corresponding to a pe/E-state tRNA within the inter-mitoribosomal subunit space. The overall positioning and domain arrangement of RRF_mt_ in the Class I map is similar to the bacterial RRF on^14,19,20,31–36^ and off^37^ the 70S ribosomes, and also to the structures of RRF bound to the 70S chloroplast ribosome^52,53^, and RRF_mt_ bound to the human mitochondrial 55S in our previous study^7^. As observed in bacteria, domain I is positioned close to the peptidyl transferase center (PTC) and extends towards the α-sarcin-ricin loop (SRL). A striking difference between the human RRF_mt_ and its bacterial counterpart is the presence of a 79 aa long N-terminal extension (NTE) in RRF_mt._ We could model the last 14 aa residues of the NTE of RRF_mt_ into an additional density contiguous with the α-helix1 from domain I. As discussed in our previous study^7^, the NTE is strategically positioned in the intersubunit space between domain I and several functionally important 16S rRNA structural elements such as H89, H90, H92 (A-loop), and MRP L16 (Fig. S4) and interacts with several nucleotides (nts) and aa residues in its vicinity^7^. Interestingly, unlike the α-helical nature inferred for the part of this segment of NTE^7^, we find that its higher resolution density to be partially unstructured. A similar observation of a relatively unstructured NTE has been recently reported in an in vivo state complex^30^. However, the mitoribosomal components interacting with RRF_mt_^7^ essentially remain unaltered.

**Fig. 2:**
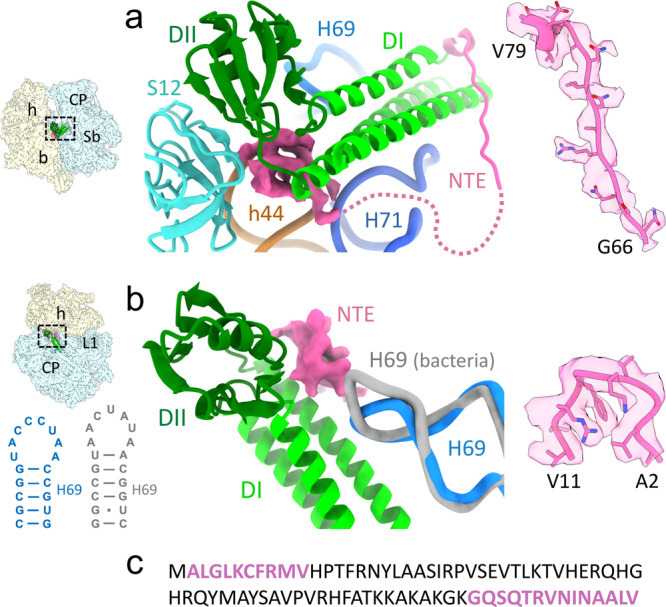
Structures of two NTE segments of RRF_mt_. **a** Density corresponding to the N-terminus segment of RRF_mt_ NTE (pink) observed in the inter-subunit space sandwiched between the 28S SSU components, including MRP uS12m (cyan) and the 12S rRNA helix h44 (brown), the 39S LSU components, including 16S rRNA helices H69 (medium blue) and H71 (dark blue), and the outer bent of the junction between domains I and II of RRF_mt_. The dotted line (pink) depicts the connection between two structurally stabilized segments of the RRF_mt_’s NTE. **b** H69 superimposition from bacterial (gray)^33^ and human mitochondrial ribosomes (blue) reveals the shortening of H69 in the mitoribosome, also depicted in secondary structures of H69 on the lower left. Domain II of RRF_mt_ interacts with the shorter H69 through its strategically positioned N-terminus of NTE (pink). **c** Sequence of NTE showing two modeled regions (pink). Thumbnails to the left depicts an overall orientation of the 55S mitoribosome, with semitransparent 28S (yellow) and 39S (blue) subunits, and overlaid positions of ligands. Landmarks on the thumbnail: h, head, and b, body of the 28S subunit, and CP central protuberance, Sb stalk base of the 39S subunit.

We found a small density in a tight pocket surrounded by the outer bend of the junction between domains I and II of RRF_mt_, MRP uS12m and the small subunit’s 12S rRNA helix h44, and the large subunit’s 16S rRNA helices H69 and H71 (Fig. 2a). Except for our previous lower resolution map of the 55S•RRF_mt_ complex^7^, this additional density is not observed in any of the available 55S mitoribosomal structures, whether complexed with other translational factors^6,44,46^ or not^3,4^. Since our complex was reconstituted from purified components, this additional density should correspond to an RRF_mt_ NTE segment, that has been stabilized through interactions with multiple mitoribosomal components in its vicinity. Though bacterial and mitochondrial ribosomes exhibit significant differences in their overall shape, composition, and conformation, their internal rRNA core regions are largely conserved^3–5,8^. Comparison of the RRF binding sites between the bacterial and mitochondrial ribosomes reveals that the H69 of 16S rRNA is slightly shorter in the mammalian mitoribosomes (Fig. 2b). This minor shortening of H69 is critical because it directly impacts the interaction of H69 with domain II of RRF_mt_. The small density, most likely corresponding to N-terminus segment of the mito-specific NTE, appears to compensate for the shortened H69 by mediating the interactions between RRF_mt_ and H69 (Fig. 2b). We generated a model that would place 10 aa residues (Ala2-Val11) at the N-terminus of NTE into this density (Fig. 2b). This model was guided by the observed sidechain density for two consecutive large side chains of Phe8 and Arg9 within the first 11 aa (Fig. 2c). Since there are other possibilities for two consecutive aa residues with large sidechains in the remaining 54 aa residues in the unmodelled segment of NTE, further confirmation of this assignment may need a well-resolved density for the entire NTE.

It should be noted that the RRF_mt_-bound 55S mitoribosomes (present work and ref. ^7^) were never observed in the unrotated state, suggesting that the RRF_mt_ binding locks the ribosome in a fully rotated state. This is in contrast to the bacterial 70S•RRF complexes that were found both in their rotated and unrotated conformational states^14,19,20,31–36^. The rotated conformation of the 55S mitoribosome seems to prime subunit dissociation by either destabilizing or completely breaking seven out of fifteen inter-subunit bridges in the unrotated 55S mitoribosome^3,8^. The simultaneous interactions of N-terminus segment of the RRF_mt_ NTE with RRF_mt_’s structurally conserved domain I, MRP uS12m, h44, H69, and H71 likely help prevent the back-rotation of the small 28S subunit. The rotated state of the 55S mitoribosome could serve as an ideal substrate for the subsequent binding of EF-G2_mt_ to complete subunit dissociation. In this context, it is important to note that the entire 79 aa long NTE is an integral part of the mature protein and is known to be essential for RRF_mt_ function during the mitoribosome recycling^29,54^.

### RRF_mt_ domain II motion helps split the 55S mitoribosome into its two subunits

Using fast-kinetics, it has been shown that the splitting/recycling of the 70S ribosome by the concerted action of RRF and EF-G happens in the sub-second time scale^22^. Both RRF and EF-G have been captured on the 70S ribosome with time-resolved cryo-EM^36^. It is more challenging without time-resolved techniques, but RRF and EF-G were also captured on the 70S ribosomes by using the factors from different species^34^ or by crosslinking the EF-G with one of the ribosomal proteins^35^. In the present work, collection of very large cryo-EM datasets (altogether 21,752 micrographs (Fig. S1), enabled the isolation of a small subset of 55S particles (Class II) that contained both RRF_mt_ and EF-G2_mt_ (Fig. 1). However, the 28 S subunit density was found to be weak and present in multiple destabilized conformations relative to the 39S subunit in this complex.

**Fig. 3:**
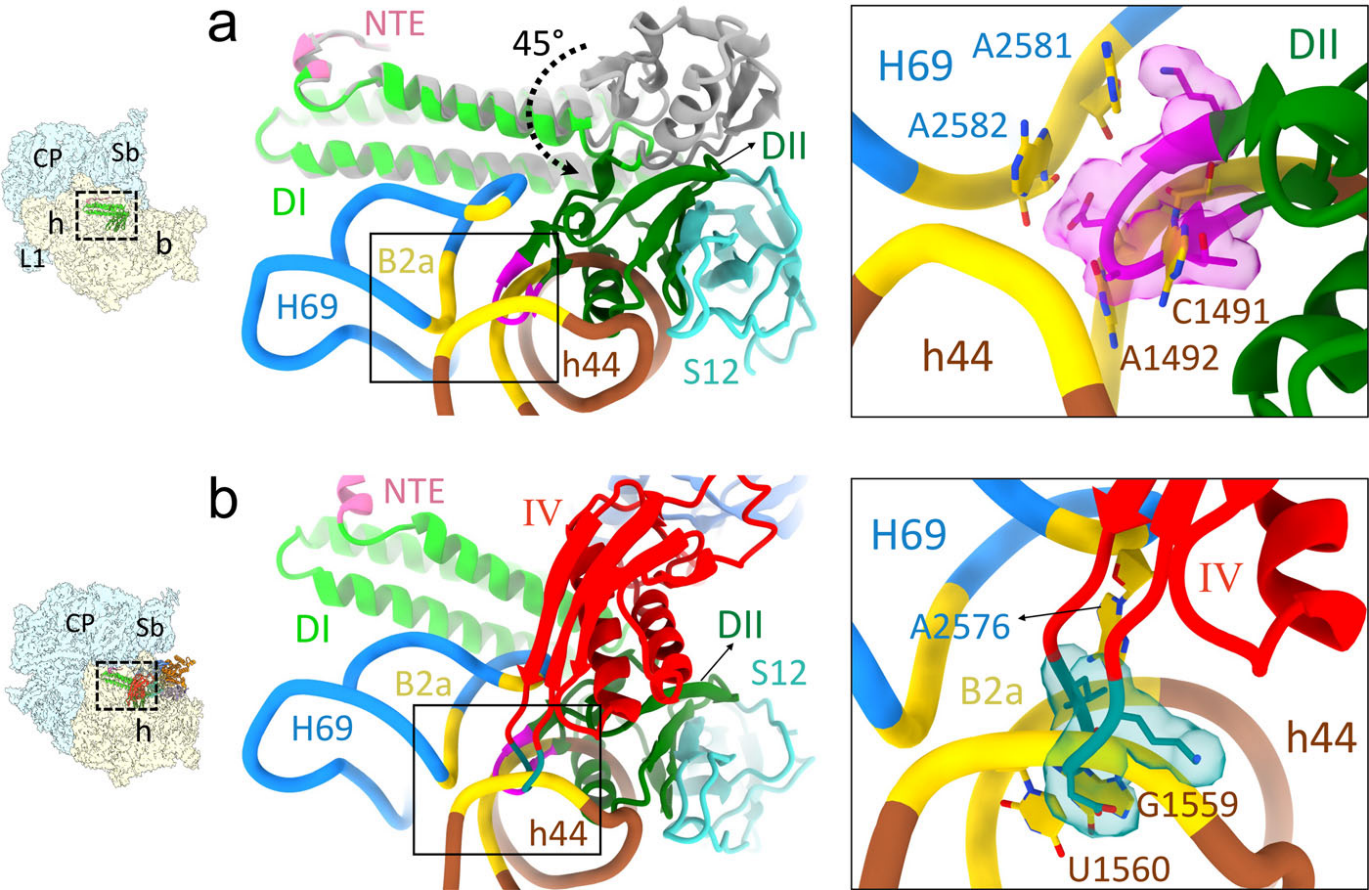
Direct involvement of RRF_mt_ domain II and EF-G1_mt_ domain IV in human mitoribosome recycling. **a** Comparison of the overall conformation of RRF_mt_ between the Class I (gray) and Class III (green) complexes revealed that domain II rotates by about 45° towards the 28S subunit. Such a rotation would sterically clash with the 12S rRNA helix 44 (h44, brown) and MRP uS12m (cyan) and destabilize a crucial inter-subunit bridge, B2a (yellow). Inset shows the magnified view of the inter-subunit bridge B2a with RRF_mt_ domain II residues V121-K127 (shown with density in magenta) disrupting B2a by inserting between h44 residues (C1491 and A1492) and H69 residues (A2581 and A2582). **b** Superimposition of the Class III complex with the Class I complex shows a direct overlap between the loop1 region (dark cyan) of EF-G2_mt_ domain IV (red) and the intersubunit bridge B2a (yellow) formed between h44 (brown) and H69 (blue). Inset shows the magnified view of EF-G2_mt_ domain IV residues L582-R585 (shown with density in dark cyan) that will disrupt the bridge B2a by inserting between h44 residues (G1559 and U1560) and H69 residue (A2576) and pushing the 28S subunit (h44) away. Thumbnails to the left depict an overall orientation of the 55S mitoribosome, with semitransparent 28S (yellow) and 39S (blue) subunits, and overlaid position RRF_mt_. Landmarks on the thumbnails are same as in Fig. 2.

Both Class II and Class III maps showed readily recognizable densities corresponding to RRF_mt_, EF-G2_mt_., and E-site tRNA. The Class III complex had superior resolution, which enabled more accurate analysis of molecular-level interactions between the two factors and the mitoribosome, and their functional implications, while the Class II map was useful for interpreting large-scale conformational changes. In line with the bacterial 70S/50S•RRF•EFG complexes^14,20,34–36^, the conformation of RRF_mt_ is substantially different between the Class I and Class III recycling complexes. The conformation of RRF_mt_ domain I remains unchanged among all three classes. In the Class II and III maps, domain II was rotated by about 45° towards the small subunit compared to its position in the Class I complex (Fig. 3a). This large conformational change is enabled by a highly flexible hinge regions between domain I and domain II in RRF_mt_. Due to this rotation, the tip of domain II moved by about 40Å towards the h44 of 12S rRNA. When the maps of Class II and III were superimposed, this motion resulted in a major steric clash between the RRF_mt_ domain II and the 28S subunit elements h44 and MRP uS12m (Fig. 3a). In the 55S mitoribosome, h44 is involved in the formation of two intersubunit bridges B2a and B3 by pairing with H69 and H71, respectively^3,8^. By displacing h44, RRF_mt_ disrupts these crucial intersubunit bridges, thereby splitting the 55S mitoribosome into its two subunits.

A well-defined 28S structure was not seen within the Class II 55S mitoribosomal complex, but several inter-subunit bridges do appear to be destabilized or broken, and the small subunit seems to be dissociating from the large subunit. To remove any possible large subunit contamination from the Class II map, extensive reference-based 3D classification was employed, but this class of 55S mitoribosomal particles with a well-resolved 39S subunit and a poorly resolved 28S subunit remained unchanged. This supports it being an ensemble of authentic, short-lived functional intermediates of mitoribosome recycling, where the small subunit is captured in multiple positions during its separation from the large subunit.

### EF-G2_mt_ binding induces conformational changes in RRF_mt_

The 55S•RRF_mt_ complex (Class I) undergoes large conformational changes upon binding to EF-G2_mt_ (Classes II and III). EF-G2_mt_ would not be able to access its binding site on the mitoribosome without the significant movement seen in domain II of RRF_mt_ in Class II and Class III complexes. This movement eliminates the direct spatial conflicts of EF-G2_mt_ domains III, IV, and V with the initial position of RRF_mt_ domain II in the Class I complex (Figs. 3a, b and Fig. S5). This change could be induced either during or upon binding of EF-G2_mt_. The domain II of RRF_mt_ is repositioned into a cavity created by domains III, IV, and V of EF-G2_mt_ (Fig. S6a) and an extensive network of interactions are formed between the two mitochondrial recycling factors. This is also in agreement with the location of RRF domain II reported in the bacterial 50S^14^ and 70S complexes^34,36^. A majority of the interactions are formed between the hinge regions that connect the two domains of RRF_mt_ and the loop regions from domain III of EF-G2_mt_. Several aa residues from the RRF_mt_ hinge region (Pro183–Thr186) interact with EF-G2_mt_ domain III residues Glu495–Leu499 (Fig. S6b) via hydrogen bonds and hydrophobic interactions. Arg187 from the α-helix following the hinge region of RRF_mt_ domain II has close hydrogen-bonding interactions with Tyr556 of EF-G2_mt_ domain III (Fig. S6b). A second set of contacts formed between the two factors involves residues Ile109–Arg110 from the second hinge region of RRF_mt_ and residues Ser527–Gln529 from the domain III of EF-G2_mt_ (Fig. S6c).

Domain IV of EF-G2_mt_ presses against domain II of RRF_mt_ through multiple interactions. The surface residues of α-helix 1 (Asn629, Ser633, and Leu636) and α-helix 2 (Thr664, Met665, Ser667, and Ala668) from domain IV of EF-G2_mt_ interact with residues in β-strand 3 (Ser134–Met138), and its adjoining loop region (Gln132 and Ile133) from domain II of RRF_mt_ through a combination of electrostatic and hydrophobic interactions (Fig. S6d). Gln637 from the α-helix 1 of EF-G2_mt_ also shares a hydrogen bond with Ser112 from the hinge region of RRF_mt_ (Fig. S6d). Contacts are observed between the C-terminal α-helix of EF-G2_mt_ domain IV and the α-helix 3 from the triple-helix bundle of RRF_mt_ domain I. In bacteria, the analogous C-terminal α-helix of EF-G is often considered as part of domain V though the first atomic models of EF-G^55,56^ grouped it with domain IV. While residues Ser776–Leu778 from EF-G2_mt_ domain IV pair with residues Arg251 and Val255 from RRF_mt_ domain I through hydrogen bonds (Fig. S6e), Arg775 from EF-G2_mt_ domain IV strongly interacts with Glu259 from RRF_mt_ domain I through a salt-bridge (Fig. S6e). Direct interactions of EF-G2_mt_ domain III with RRF_mt_ domain II at its hinge regions, known to confer interdomain flexibility to the bacterial factor^38^, likely help trigger the dissociation of 55S mitoribosomes into subunits by enabling the repositioning of RRF_mt_ domain II. At the same time, the multiple interactions between domain IV of EF-G2_mt_ and domain II of RRF_mt_ appear to stabilize the RRF_mt_ domain II in the altered position, which would push the 28S subunit away from the 39S subunit and prevent domain II from reverting back to its previous orientation, in order to maintain the 39S•RRF_mt_•EF-G2_mt_ complex in a dissociated state.

In addition to aiding to the function of RRF_mt_, EF-G2_mt_ plays a direct role in destabilizing the 55S mitoribosome. Superimposition of the maps of Class I and Class III complexes reveals a direct steric clash between the loop1 region of EF-G2_mt_ domain IV and the 28S subunit component (rRNA h44), that participates in the formation of the inter-subunit bridge B2a (Fig. 3b). More importantly, the orientation of domain IV loop1 seems to be unique to EF-G2_mt_ since the analogous region in EF-G1_mt_ is positioned away from the intersubunit bridge B2a towards the decoding center^6,46^.

### Role of mito-specific MRPs in stabilizing the deacylated tRNA

**Fig. 4:**
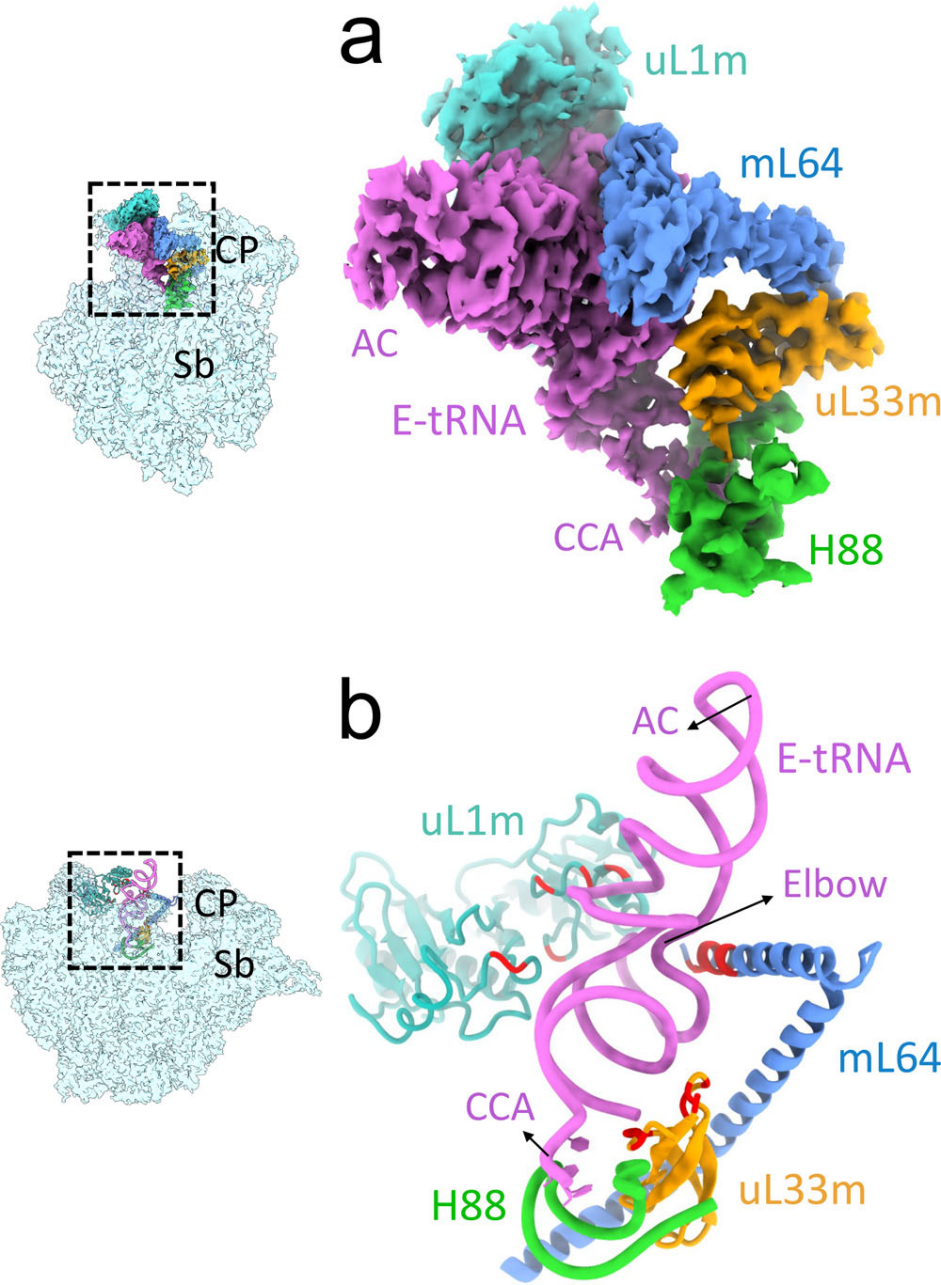
Stabilization of the E-site tRNA on the dissociated 39S subunit. **a** Extracted cryo-EM densities corresponding to E-site tRNA and interacting 39S subunit components. The E-site tRNA (orchid) is stabilized on the 39S subunit through multiple interactions with the mitoribosomal components. The elbow region of the E-site tRNA is sandwiched between 39S MRPs uL1m (cyan) and the mitospecific mL64 (blue) while the acceptor arm and the CCA end have strong interactions with uL33m and the 16S rRNA helix H88, respectively. **b** Atomic models for the densities shown in **a**. Multiple MRP segments bearing positively charged aa residues (Arg and Lys; red) interact with the negatively charged phosphates of the E-site tRNA molecule. To better display the location of the positively charged aa residues at the interface of tRNA and MRPs, the **b** orientation is obtained by applying an upward rotation around a horizontal axis to the panel **a** orientation. AC and CCA refer to anticodon and CCA ends of the tRNA. Thumbnails to the left depict an overall orientation of the 39S subunit (semitransparent blue), with overlaid highlighted mitoribosomal components and ligands. Landmarks on the thumbnail: CP central protuberance, Sb stalk base.

The presence of E-site tRNA in Class III complex of the dissociated 39S subunit suggests a markedly different mechanism of deacylated tRNA removal during recycling of mitochondrial PoTC as compared to that in the bacteria, where the deacylated tRNA goes with the small ribosomal subunit during the 70S ribosome splitting^36^. This observation is particularly intriguing since a majority (11 of 12) of the rRNA segments in bacterial 23S rRNA that are known to interact with the E-site tRNA are absent in the mito-16S rRNA^57^. We find that the E-site tRNA in the 39S complex is stabilized by multiple interactions with Arg-rich and Lys-rich electro-positive segments of MRPs, including mL64^6^, uL33m, and uL1m^58^ (Fig. 4). With the tightly held body of the deacylated tRNA through conserved interaction of its CCA end with the rRNA H88 and multiple new interactions with MRPs, transition of its anticodon end from a pe/E state^7^ in Class I complex to E/E-state in Class II and III complexes could in part facilitate the dissociation of the two mitoribosomal subunits. Moreover, the mechanism of subsequent release of deacylated tRNA from the 39S subunit remains unknown.

### Dynamic interactions among uL11m, CTD of uL12m, and EF-G2_mt_

**Fig. 5:**
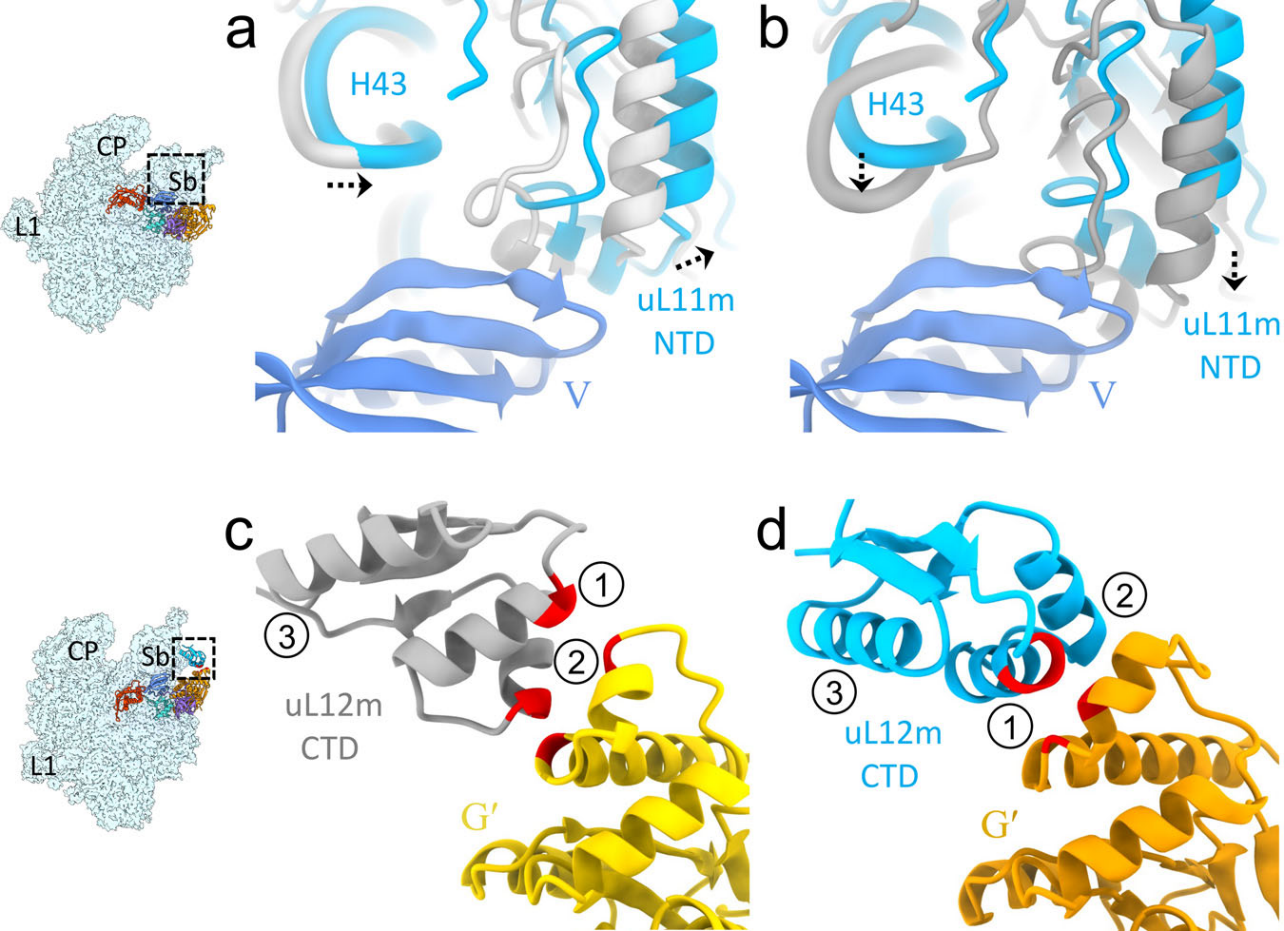
EF-G2_mt_ binding induces large-scale conformational changes in the uL11m stalk-base region and the CTD of uL12m. **a** In the 39S•RRF_mt_•EF-G2_mt_ complex, the uL11m stalk-base region (blue) moves towards the CTD of uL12m and away from the domain V of EF-G2_mt_, as compared to its position in the EF-G2_mt_-unbound 55S mitoribosome (light gray)^3^. **b** In sharp contrast, in the presence of EF-G1_mt_, the uL11m stalk-base region was found to move towards the domain V of EF-G1_mt_ in the translocation complex (dark gray)^6^. **c** In the 55S•EF-G1_mt_ complex^6^, the CTD of uL12m (gray) is positioned in such a way that its α-helices 1 and 2 directly interact with the G′ subdomain of EF-G1_mt_ (yellow). **d** In our 39S•RRF_mt_•EF-G2_mt_ complex, the CTD of uL12m (blue) is rotated by about 60° and shifted away by about 7 Å from the G′ subdomain of EF-G2_mt_, resulting in the loss of contacts between its α-helix 2 and G′ subdomain of EF-G2_mt_ while a new set of interactions is formed between its α-helix 1 and G′ subdomain of EF-G2_mt_. Thumbnails to the left depict an overall orientation of the 39S subunit (semitransparent blue), and overlaid positions of the ligands. Landmarks on the thumbnail: CP central protuberance, Sb stalk base, L1 MRP uL1m.

EF-G2_mt_ binding resulted in a prominent conformational change in the uL11m stalk-base region of the 39S subunit. The uL11m stalk-base region moved towards the CTD of uL12m, a component of the L10–L12 stalk, and assumed a unique conformation not reported previously^3,4,6,7,44^. The 16S rRNA H43 of the uL11m stalk-base moved by 3 Å towards the CTD of uL12m, oriented parallel to the domain V of EF-G2_mt_, while the NTD of MRP uL11m moved about 5 Å away from the EF-G2_mt_ domain V (Fig. 5a). This is in sharp contrast to the 55S•EF-G1_mt_ translocation complexes^3,4,6,7,44,46^, where the uL11m stalk-base region was observed to move 5 Å closer towards the domain V of EF-G1_mt_ (Fig. 5b). The movement of uL11m away from the EF-G2_mt_ domain V and towards the CTD of uL12m is essential for the binding of EF-G2_mt_ in the present conformation, to avoid the steric clash between the NTD of uL11m and domain V of EF-G_mt_ (Fig. 5a). It is also possible that the conformation of EF-G2_mt_ observed in our 39S•EF-G2_mt_ complex (Class III) was attained after the dissociation of 39S subunit from the 55S complex (Class II).

In addition to the unique conformation of the uL11m stalk-base region, the CTD of uL12m was also observed in a distinct conformation. In the 55S•EF-G1_mt_ complexes^6,46^, the CTD of uL12m is positioned close to EF-G1_mt_ so that α-helices 1 and 2 of uL12m CTD would have close interactions with the G′ subdomain of EF-G1_mt_ (Fig. 5c), thereby providing stability to the otherwise flexible uL12m CTD. In contrast, the CTD of uL12m rotates by about 60° and shifts away by about 7 Å from the G′ subdomain of EF-G2_mt_ in the 39S•EF-G2_mt_ complex (Fig. 5d). As a result of this large rotational movement, interactions between the uL12m CTD α-helix 2 and the G′ subdomain of EF-G2_mt_ are lost, while the contacts between the α-helix 1 and the G′ subdomain of EF-G2_mt_ are maintained (Fig. 5d). Since protein uL12 is known to play a central role in the recruitment of translational factors to the bacterial ribosome^59–62^; the semi-stable conformation of uL12m observed in the 39S•EF-G2_mt_ complex represents a late-stage conformation of uL12m prior to its detachment from the EF-G2_mt_ as EF-G2_mt_’s participation in the 55S ribosome recycling process nears completion.

### Structural basis for use of EF-G2_mt_ in mitoribosomal recycling instead of EF-G1_mt_

**Fig. 6:**
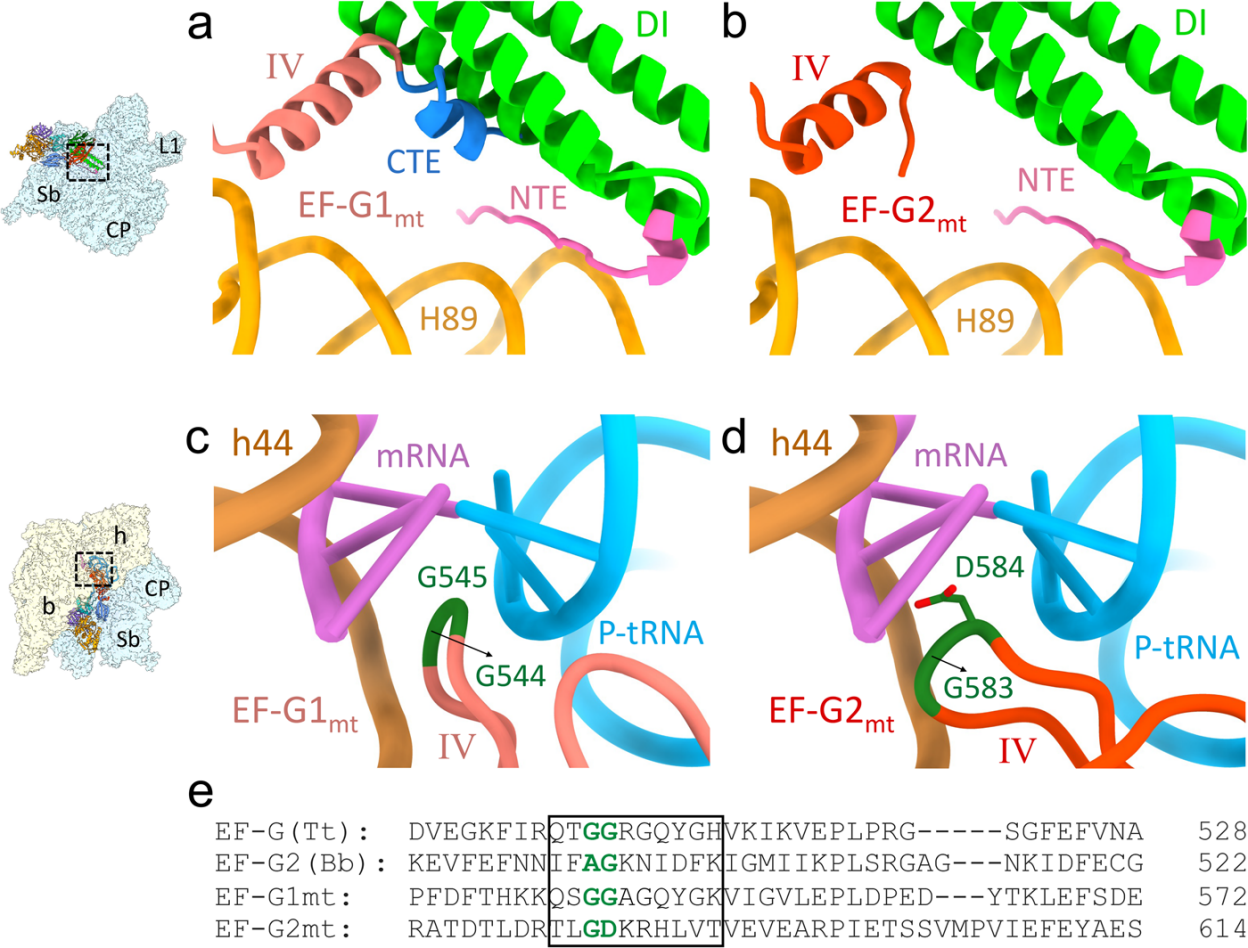
Structural basis for the exclusive roles of EF-G1_mt_ in tRNA translocation and EF-G2_mt_ in mitoribosome recycling. **a** The presence of a substantially longer C-terminal α-helix (salmon) along with the presence of a unique CTE (blue), which is required for mitochondria tRNA translocation^6^, prevents the simultaneous binding of EF-G1_mt_ and RRF_mt_ (green) on the mitoribosome due to a major steric clash. Furthermore, any conformational repositioning the C-terminal α-helix of EF-G1_mt_ will result in direct steric clash with the H89 (orange) of 16S rRNA and the NTE of RRF_mt_ (pink)**. b** Having a shorter C-terminal α-helix (red) and the absence of CTE allows the simultaneous binding of EF-G2_mt_ and RRF_mt_ (green) on the mitoribosome. **c** In EF-G1_mt_^6^, the presence of two universally conserved glycine residues (dark green) at the tip of domain IV loop1 region (salmon) facilitates its insertion between the mRNA-tRNA duplex at the decoding center (DC) during EF-G1_mt_-catalyzed translocation. **d** In EF-G2_mt,_ the second glycine of domain IV loop 1 region (red) is substituted by an aspartic acid (dark green) altering its conformation and flexibility, and thereby making its insertion into the DC during translocation unfavorable. **e** The boxed aa sequence corresponds to the loop 1 situated at the tip of domain IV, which is conserved between EF-G1_mt_ and the *T. thermophilus* EF-G. First of the two universally conserved loop 1 glycine residues (green) is substituted by an alanine in *B. burgdorferi* EF-G2, while the second one is replaced by an aspartic acid in EF-G2_mt_.

In most bacterial species, a single EF-G is involved in both translocation and ribosome recycling. Mammalian mitoribosomes have evolved to utilize the two isoforms EF-G1_mt_ and EF-G2_mt_ to cope with the significantly altered environment in mitochondria as compared to the bacterial cytoplasm^26,27^ and perform two separate functions^26,27^. EF-G1_mt_ is used during translation elongation while EF-G2_mt_ is used along with RRF_mt_ for the recycling of the 55S mitoribosome. The overall position and domain arrangement of EF-G2_mt_ is similar to that of EF-G1_mt_ in the recently published human^6^ and porcine^46^ translocational complexes (Fig. S6). There are, however, some specific structural distinctions between the two factors that assign them specialized functional roles. The most striking of these is the presence of a C-terminal extension (CTE) in EF-G1_mt_ domain IV (Fig. 6a)^6^. Besides its CTE, the size of the conserved C-terminal α-helix of EF-G1_mt_ domain IV is substantially longer (16 aa) (Fig. 6a) than the C-terminal α-helix (12 aa) of EF-G2_mt_ domain IV (Fig. 6b).

The sturdier and longer C-terminal α-helix of EF-G1_mt_ domain IV and its 11 aa CTE would not permit the coexistence of RRF_mt_ on the mitoribosome due to a major steric clash between domain I of RRF_mt_ and the C-terminal region of EF-G1_mt_ (Fig. 6b). Even a reorientation of the C-terminal α-helix and its CTE away from the domain I of RRF_mt_ would not resolve the problem as they would then clash with H89 of the 16S rRNA and the NTE of RRF_mt_, that has been positioned in the inter subunit space between the domain I of RRF_mt_ and 16S rRNA helix, H89 (Fig. 6b). Structural analysis of the bacterial EF-Gs from various species^47,63,64^ has revealed that the length of their C-terminal α-helices are about 12 aa long, suggesting that only EF-G2_mt_ can function alongside RRF_mt_ during subunit splitting. Interestingly, the C-terminal α-helices of EF-G2 from *T. thermophilus*^39^ and EF-G2_mt_ are similar in size (Fig. S7) though the function of EF-G2 in *T. thermophilus* is not understood. Moreover, the interaction between the C-terminal regions of EF-G2_mt_ domain IV and RRF_mt_ domain I is essential for stabilizing the bound RRF_mt_. This tight anchoring of RRF_mt_ domain I to the mitoribosome would prevent RRF_mt_ dissociation from the mitoribosome, when its domain II undergoes substantial rotation to displace the h44 region of the 28S subunit. This agrees with the observation that deletion of the last few aa residues from the C-terminal region of bacterial RRF adversely effects its function during 70S ribosome recycling^65^.

Now the question is why EF-G1_mt_ mediates the translocation step during mitoribosomal elongation and not EF-G2_mt_? The most probable answer is that the CTE in the domain IV of EF-G1_mt_ that limits its ability to participate in the mitochondrial ribosome recycling, is directly involved in the elongation step of mammalian mitochondrial protein synthesis^6^. By contacting the inner bend of the A-site tRNA, the CTE of EF-G1_mt_ can help in translocating the CCA arm of the A-site tRNA into the P site, and by interacting with a 16S rRNA helix, H71, it prevents the P-site tRNA from reverting back into the A site^6^. This analysis is further supported by the fact that bacterial EF-Gs, which lack the CTE, are inactive on the 55S mitochondrial ribosomes while EF-G1_mt_ is active on the 70S ribosomes^66^.

Specific sequence differences in a critical region (loop1) within the domain IV of EF-G2_mt_ can also make it ineffective in driving translocation. During EF-G-catalyzed translocation in bacteria, the presence of two universally conserved glycine residues at the tip of domain IV (loop 1) region facilitate the insertion of domain IV into the decoding center (DC)^47^. In the DC, the loop 1 of domain IV destabilizes the codon–anticodon interactions of the mRNA-tRNA duplex with the universally conserved 16S rRNA bases A1492 and A1493 in the 30S A site thereby aiding the A-site tRNA along with its associated codon to translocate into the P site^67^. The loop 1 region is conserved in EF-G1_mt_ but is significantly altered in EF-G2_mt_ (Fig. 6e). EF-G1_mt_ retains both the glycine residues (Gly544 and Gly545) in its domain IV loop 1 region (Fig. 6c) whereas the second glycine is replaced by an aspartic acid in EF-G2_mt_ (Fig. 6d), which alters the conformation of the tip of domain IV rendering it structurally unfavorable for insertion into the grove between the P-site tRNA and the associated codon (Fig. 6d). A similar observation has been made in case of the spirochaete *B. burgdorferi* that utilizes EF-G2 exclusively for ribosome recycling^42^, where one of these conserved loop1 glycines is substituted by an alanine (Fig. 6e) rendering it inactive for promoting the translocation step. Interestingly, however, the *T. thermophilus* EF-G2 carries both the conserved glycines. Moreover, the Ala546 and Gly547 residues that follow the conserved glycines of loop 1 in EF-G1_mt_ are substituted by the large polar sidechain residues lysine and arginine, respectively, in the corresponding region of EF-G2_mt_ (Fig. 6e), thereby significantly altering the hydrophobicity of the loop 1 tip. Point mutations and deletions at the loop 1 tip region are known to have a pronounced effect on the function of EF-G during the elongation step of bacterial protein synthesis^68^, but have a negligible effect on the activity of EF-G in bacterial ribosome disassembly^69^. Our study thus provides a structural rationale for the biochemical finding that mammalian mitoribosomes utilize two distinct EF-G-like factors during the translation elongation and recycling phases^27^.

### Mammalian 55S mitoribosome recycling does not require GTP hydrolysis by EF-G2_mt_

The overall G domain structure in the 39S•RRF_mt_•EF-G2_mt_ complex (Class III map) is similar to the G domain in the 55S•EF-G1_mt_ complex^6,46^. The well-ordered density in our maps of highly conserved translational GTPase consensus motifs such as the P-loop, switch I and switch II regions, allowed complete modeling of these essential regions. A well-defined density corresponding to a bound GMPPCP molecule is also readily identifiable in the nucleotide binding pocket. GMPPCP is stabilized through interactions with universally conserved aa residues, such as Asp80 and Lys83 of P-loop, Thr122 of switch I and His145 of switch II (Fig. S8a). As in the 55S•EF-G1_mt_ complex^6^, a crucial Mg^2+^ ion is positioned near the γ phosphate of GMPPCP and is coordinated by Thr84 and Thr122 from the P-loop and switch I regions, respectively (Fig. S8a). The catalytic His145 (His124 in EF-G1_mt_), known to play a central role in the hydrolysis of the bound nucleotide^70,71^, is found oriented towards the γ phosphate of the bound GMPPCP (Fig. S8a), suggesting an active conformation of the factor prior to GTP hydrolysis.

The highly conserved α-sarcin-ricin stem-loop (SRL) region is known to be essential for the GTPase activity of all the translational G proteins^47,70,71^. Base A3129 from the SRL was found to be contacting the Switch II His145 through hydrogen bonding interactions and thereby stabilizing His145 in its activated conformation poised to perform the hydrolysis reaction (Fig. S8b). While EF-G-dependent GTP hydrolysis is essential for an efficient splitting of the 70S ribosome into its subunits^16,18,22^, dissociation of the mammalian 55S mitoribosomes does not require EF-G2_mt_-dependent GTP hydrolysis, which is only needed for the release of EF-G2_mt_ from the dissociated large subunit^27^. Our structural results strongly corroborate the Tsuboi and coworkers^27^ observation as a significant proportion (82%) of the EF-G2_mt_ was found complexed to the 39S subunits as compared to the small proportion (18%), that remained associated with the 55S mitoribosomes (see Fig. S1). Even though GTP hydrolysis by EF-G2_mt_ is not necessary for 55S mitoribosome splitting, the presence of GTP or its non-hydrolysable analogs GDPNP/GMPPCP in the nucleotide binding pocket is essential, as the presence of GDP or the absence of any nucleotide does not split the 55S mitoribosome^27^.

### Divergent mechanisms of fusidic acid (FA) resistance by EF-G1_mt_ and EF-G2_mt_

**Fig. 7:**
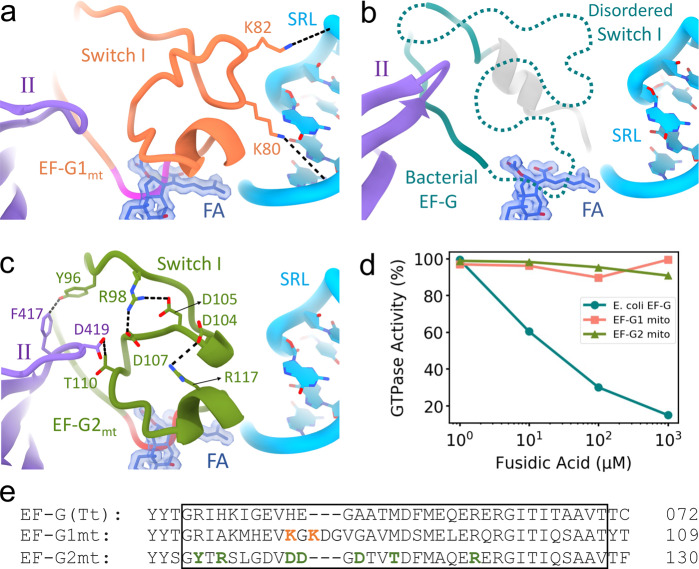
EF-G1_mt_ and EF-G2_mt_ follow diverse mechanisms to render resistance to the antibiotic fusidic acid (FA). **a** Stabilized Switch I region (salmon)^6^ in EF-G1_mt_ blocks FA (dark blue) from accessing its binding site. The Switch I region sterically overlapping with FA is colored in magenta. Switch I stability is achieved by the presence of two unique lysine residues (K80 and K82) that strongly interact with the phosphates of SRL by forming salt bridges (light blue)^46^. **b** The absence of these lysine residues in the Switch I region (dark cyan) of bacterial EF-G disorders the Switch I^47^ and hence makes it susceptible to FA binding and inhibition. **c** In EF-G2_mt_, the primary aa sequence of switch I (green) is highly altered enabling the formation of three salt bridges within its switch I, thereby stabilizing it. Furthermore, strong interactions are observed between the switch I and domain II (purple) in EF-G2_mt_. The Switch I region sterically overlapping with FA is colored in red. **d** EF-G1_mt_ (salmon) and EF-G2_mt_ (green) show strong resistance even at high concentrations of FA, while the bacterial EF-G (dark cyan) is susceptible to FA inhibition even at low concentrations. Each point in the plots represents the average of normalized data from two independent sets of experiments. Raw luminescence values obtained from the GTPase-Glo™ Assay are shown in Supplementary Table 1. **e** The aa sequence alignment of the switch I region (boxed) in three EF-Gs. The lysine residues shown in salmon are unique to mammalian EF-G1_mt_ and confer stability to switch I by interacting with the SRL. Residues shown in green (except R117) are unique to EF-G2_mt_ and confer stability to switch I by forming salt bridges within the switch I region.

FA is a fusidane class antibiotic that is used to treat bacterial skin infections along with chronic bone and joint infections. It is effective against several species of gram-positive bacteria and is clinically used to treat methicillin-resistant *S. aureus* (MRSA). FA prevents the release of EF-G•GDP from the 70S ribosome after GTP hydrolysis by preventing the switch II from attaining its GDP-bound conformation^47^. Prior structural studies have demonstrated that FA binds in an interdomain pocket between the G domain, domain II, and domain III of EF-G^47,72^. Stable binding of FA requires the switch I region to be disordered^47^, because an ordered switch I would overlap with the binding site of FA. Biochemical studies have shown that a substantially higher concentration (10-fold to 100-fold) of FA is needed to inhibit the activity of EF-G1_mt_ during mitochondrial elongation^28,73^. Recent cryo-EM structures of EF-G1_mt_ bound to the 55S mitoribosomes have presented the switch I in a well-defined conformation^6,46^ (Fig. 7a), in contrast to bacterial 70S•EF-G complexes where the density for switch I has been consistently poorly resolved (Fig. 7b)^47,67,70–72^. It was proposed that the increased resistance observed for EF-G1_mt_ towards FA resulted from a small insertion in the switch I region of EF-G1_mt_^46^. The two positively charged lysine residues (Lys80 and Lys82) in this insertion form salt bridges with the negatively charged phosphate backbone of the SRL from the 39S subunit (Fig. 7b), and hence were hypothesized to confer additional stability to the switch I of EF-G1_mt_^46^. Biochemical evidence showed EF-G1_mt_ to be more resistant towards FA than its bacterial counterpart^28,73^.

FA is known to inhibit both the translocation and the ribosome recycling steps in bacteria, though there is conflicting evidence on which step of translation is primarily targeted by FA^22,25^. Comparison of the FA binding pocket between the bacterial EF-G, EF-G1_mt_, and EF-G2_mt_ revealed that key aa residues (Fig. S9a) reported to be necessary for the stable binding of FA^47,72^ are highly conserved (Fig. S9c), thereby suggesting a similar binding mechanism for FA for all the three EF-Gs. The three aa insertion in EF-G1_mt_ that confers resistance to FA is not present in EF-G2_mt_ and the corresponding region in EF-G2_mt_ does not contain any positively charged aa residues (Fig. 7e), that could strongly interact with the SRL. However, the cryo-EM map of EF-G2_mt_ shows a well-resolved density for the switch I region (Fig. S9b) and enabled its modeling (Fig. 7c), indicating an alternative mechanism for the stabilization of switch I in EF-G2_mt_. Sequence alignment showed that the switch I region composition in EF-G2_mt_ is significantly different as compared to EF-G1_mt_ and the bacterial EF-G (Fig. 7e). Three new salt bridge interactions were identified within the switch I of EF-G2_mt_. Arg98 forms the first two salt bridges by pairing with Asp105 and Asp107, respectively, while Arg117 and Asp104 are involved in the formation of the third salt bridge (Fig. 7c). Furthermore, stronger interactions are observed between the switch I and domain II in EF-G2_mt_ compared to EF-G1_mt_. Thr110 from switch I is placed in the close proximity of Asp419 of domain II with the possibility of hydrogen bond formation (Fig. 7c), while the corresponding interaction in EF-G1_mt_ is between Asp404 and Val88, a much weaker interaction with no possibility of hydrogen bond formation. There is also potential for a tight T-stacking interaction between Phe417 and Tyr96 in EF-G2_mt_ (Fig. 7c), while the corresponding residues in EF-G1_mt_ being His402 and Arg72 offer no such interaction. Overall, through a combination of internal salt bridges and additional contacts with domain II, switch I gets highly stabilized in EF-G2_mt_. Since a stabilized switch I region occludes the binding site of FA (Fig. 7a, b), EF-G2_mt_ is expected to exhibit strong resistance towards FA in the lines of EF-G1_mt_. To test this hypothesis, we measured the GTPase activity of EF-G2_mt_ alongside EF-G1_mt_ and *E. coli* EF-G in the presence of FA under multiple-turnover conditions (see Methods). Our data shows that while 1 µM FA has no effect on the GTPase activity in *E. coli*, significant inhibition is observed at higher concentrations of FA. In contrast, FA has almost no effect on the GTPase activity of either EF-G1_mt_ or EF-G2_mt_ even up to 10 mM FA. Our results are consistent with the earlier finding that EF-G1_mt_ is highly resistant to FA compared to the bacterial EF-G^28,73^, and also consistent with recent finding that showed EF-G2_mt_ is not susceptible to inhibition by FA^74^. Our results and analysis suggest FA resistance in EF-G2_mt_ occurs by a structural mechanism fundamentally different from that in EF-G1_mt_.

**Fig. 8:**
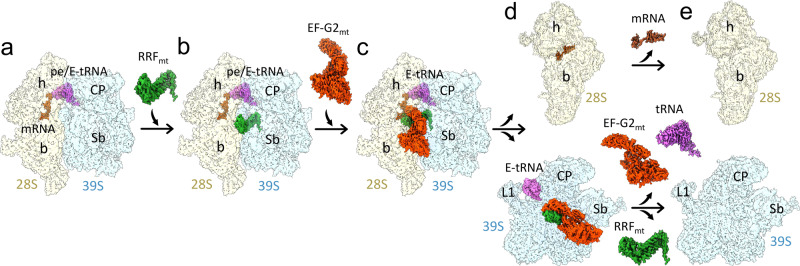
Sequence of main events in human mitochondrial ribosome recycling suggested by structures in this study. **a** The model post-termination mitoribosomal complex (PoTC), with mRNA and pe/E-state tRNA_mt_ (Koripella et al.^43^). **b** Binding of RRF_mt_ locks the mitoribosome in a partially destabilized state with rotated 28S subunit (Figs. 1a and S3b). The mito-specific N-terminus of the RRF_mt_’s NTE occupies a unique site near the bridge B2a region (Fig. 2). **c** Subsequent binding of EF-G2_mt_ further destabilizes the 55S complex with disruption of additional inter-subunit bridges, in a fast reaction leading to multiple short-lived intermediate states, with the 28S subunit present in multiple orientations relative to the 39S subunit (Fig. 1b). **d** 28S and 39S subunits are dissociated. While the 28S subunit still carries mRNA, the 39S subunit carries RRF_mt_, EF-G2_mt_, and surprisingly a deacylated tRNA_mt_ in the 39S subunit’s E-site region (Figs. 1c and 4). This is in sharp contrast to the tRNA dissociation mechanism in eubacterial ribosome recycling, where tRNA goes with the small 30S ribosomal subunit. **e** Depiction of final recycling products, steps of ligand release between **d** and **e** are yet to be characterized.

In conclusion, structures of three distinct functional states formed during the process of human mitoribosome recycling are presented (Fig. 1). A previous biochemical finding that GTP hydrolysis is not required for the RRF_mt_•EF-G2_mt_-mediated splitting of the post-termination mitoribosomal complex is corroborated. We also show that a mito-specific segment of the RRF_mt_’s NTE compensates for the slightly reduced size of the H69 within the 16S rRNA of the mitoribosomal large subunit (Fig. 2). This is the first evidence showing a translational factor compensating for an rRNA segment lost, perhaps during the evolution of the mammalian mitochondrial translation machinery. Our structures reveal how RRF_mt_’s domain II and EF-G2_mt_ domain IV directly help in disrupting the central inter-subunit bridge, B2a (Fig. 3), how the deacylated tRNA is held with the dissociated 39S subunit components (Fig. 4), and suggest how the dynamics of the interactions among the uL11m, CTD of uL12m, and the G’domain of EF-G2_mt_ alters between elongation and recycling steps (Fig. 5). Structural analysis of domain IV of EF-G1_mt_ and EF-G2_mt_ explains their specific roles in two distinct steps of elongation and recycling, respectively (Fig. 6). Analysis of their GTPase domains complemented by GTPase assays reveal two distinct mechanisms of antibiotic fusidic acid resistance adopted by two homologous GTPases (Fig. 7). These observations allow us to highlight distinct features of the main steps of human mitoribosomal recycling (Fig. 8). Future studies using time-resolved cryo-EM should help further resolve the short-lived intermediates that form during the transition from Class I to Class III states.

## Methods

### Purification of 55S mitoribosomes

The source of mitochondrial ribosomes was human embryonic kidney cells lacking N-acetyl-glucosaminyltransferase I (HEK293S GnTI) that were cultured in roller bottles using FreeStyle^TM^293 media (Gibco, Life Technologies) supplemented with 5% fetal bovine serum (Gibco, Life Technologies). After centrifugation at 1000 × *g* for 7 min, the HEK293S GnTI cell-pellet was transferred to a glass homogenizer and resuspended in buffer containing 50 mM HEPES-KOH pH 7.5, 10 mM KCL, 1.5 mM MgOAc, 70 mM sucrose, 210 mM mannitol, 1 mM EDTA, 1 mM EGTA, 1 mM DTT, and 1 mM PMSF. The cells were homogenized by applying 120 strokes and the supernatant was separated from the cell debris by spinning at 950 × *g* for 15 min. The supernatant was then spun at 11,000 × *g* for 15 min, and the resulting pellet that contains crude mitochondria was resuspended in SEM buffer (250 mM sucrose, 20 mM HEPES-KOH pH 7.5, 1 mM EDTA, and 1 mM EGTA). RNase-free DNase **I** (3 units/ml) was added to the crude mitochondria and incubated at 4 °C for 1 h in a rocking platform to allow gentle mixing. A discontinuous gradient was prepared in a Beckman polyallomer tube by layering 2.5 ml of 60%, 4 ml of 32%, 1 ml of 23%, and 1 ml of 15% sucrose solutions in buffer containing 10 mM HEPES-KOH pH 7.5 and 1 mM EDTA. DNase-treated sample was loaded on the discontinuous gradient and centrifuged for 1 h at 135,000 × *g* using Ti70 rotor in Beckman ultracentrifuge. The brownish-orange layer containing pure mitochondria was carefully separated and re-suspended in SEM buffer.

Four volumes of lysis buffer (25 mM HEPES-KOH pH 7.5, 100 mM KCl, 25 mM MgOAc, 1.7% Triton X-100, 2 mM DTT and 1 mM PMSF) was added to the mitochondrial-pellet and then incubated for 15 min at 4 °C. The sample was centrifuged at 30,000 × *g* for 20 min and the supernatant was loaded on top of 1 M sucrose cushion in buffer (20 mM HEPES-KOH pH 7.5, 100 mM KCl, 20 mM MgOAc, 1% Triton X-100 and 2 mM DTT). After centrifugation for 17 h at 90,000 × *g* using Ti70 rotor in Beckman ultracentrifuge, a minimal volume of Mitobuffer (20 mM HEPES-KOH pH 7.5, 100 mM KCl, 20 mM MgOAc, and 2 mM DTT) enough to dissolve the pellet was added. 10–30% continuous sucrose density gradients were prepared in Mitobuffer, using the gradient making apparatus (C.B.S. Scientific Co.). The resuspended pellet was subjected to 10–30% continuous sucrose density gradient centrifugation at 60,000 × *g* for 17 h using Sw32 rotor in Beckman ultracentrifuge. The gradient was fractionated on ISCO gradient analyzer (Teledyne ISCO, Inc), and the fractions corresponding to the mitoribosomes were collected and pooled. Finally, the pooled mitoribosomes were concentrated by spinning them at 130,000 × *g* for 6 h using Ti70 rotor, and the pellet was resuspended in Polymix buffer (5 mM HEPES-KOH pH 7.5, 100 mM KCl, 20 mM MgOAc, 5 mM NH_4_Cl, 0.5 mM CaCl_2_, 1 mM DTT, 1 mM spermidine, and 8 mM putrescine).

### Overexpression and purification of RRF2_mt_

RRF_mt_ clone was a gift from Prof. Linda Spremulli, University of North Carolina. The SUMO-tagged RRF_mt_ was over-expressed in Rosetta2 cell lines and lysis buffer (500 mM NaCl, 1× PBS, 4 mM ß-mercaptoethanol, 1 mM PMSF, and 10 mM imidazole) was added to the pelleted cells. After sonication, the lysate was treated with DNase and then centrifuged for 30 min at 16,000 × *g*. The supernatant was applied to a His-trap Ni^2+^ column and the SUMO-tagged protein was eluted from the column using elution buffer (250 mM NaCl, 1× PBS, 4 mM ß-mercaptoethanol, and 300 mM imidazole) using standard affinity purification protocols. The purified protein was dialyzed in buffer (20 mM Tris-HCl pH 8.0, 250 mM NaCl, 4 mM ß-mercaptoethanol, and 5% glycerol) and then the SUMO tag was cleaved from RRF_mt_ by incubation with SUMO express protease for 1 h at 30 °C. Finally, the SUMO tag and the SUMO express protease were separated from the RRF_mt_ by passing through a His-trap Ni^2+^ column that specifically adsorbs the SUMO tag and the SUMO express protease while pure RRF_mt_ was released into the column flow-through.

### Overexpression and purification of EF-G2_mt_

The GST-tagged EF-G2_mt_ was cloned into pGEX6.1 vector and over-expressed in Rossetta (pLysS + RARE) cell lines. Cells were grown in LB media with 100 µg/ml ampicillin until 0.6 O.D. and protein over-expression was induced by adding 100 mM isopropyl-1-thio-d-galactopyranoside (IPTG). The cell culture was left overnight at 16 °C for optimal protein yields. The cells were pelleted in a JLA 10.5 rotor at 5000 rpm for 30 min and were shock-frozen in liquid nitrogen and stored at −80 °C. The frozen cells were resuspended in lysis buffer (1× PBS-pH7.5, 10 mM MgCl_2_, 1 mM phenylmethyl sulphonyl fluoride (PMSF) and 1 mM DTT). After sonication and DNase I (5 μg/ml) treatment, the lysate were centrifuged at 14,000 rpm for 30 min to remove cell debris. The supernatant was passed through GSTrap™ HP column equilibrated with binding buffer 1× PBS-pH7.5 and 1 mM DTT. The GST-tag was cleaved from EF-G2_mt_ by loading 3C precision protease (gifted by Dr. Hongmin Li’s lab, Wadsworth center, USA) along with cleavage buffer (50 mM Tris-HCl-pH 7.5, 150 mM NaCl, and 1 mM DTT). After incubating the EF-G2_mt_ with 3C precision protease overnight, relatively pure EF-G2_mt_ was eluted by passing the elution buffer (50 mM Tris-HCl-pH 7.5, 150 mM NaCl and 1 mM DTT). To obtain high-level purity, EF-G2_mt_ was further passed through an anion exchange HiTrap Q HP column (GE healthcare, USA) and pure protein was eluted by running a gradient with 20 mM Tris-HCl-pH 7.5 and 500 mM NaCl.

### Preparation of the 55S•RRF_mt_•EF-G2_mt_•GMPPCP complex

Fifty micromolar of puromycin and 150 nM 55S mitoribosomes were mixed in HEPES polymix buffer (5 mM HEPES-KOH pH 7.5, 100 mM KCl, 20 mM MgOAc, 5 mM NH_4_Cl, 0.5 mM CaCl_2_, 1 mM DTT, 1 mM spermidine, and 8 mM putrescine) and incubated for 10 min at 37 °C to obtain the model post-termination (PoTC) complex. Fifteen micromolar of RRF_mt_ was added to this reaction mixture of PoTC and incubated for an additional 5 min at 37 °C to obtain the 55S•RRF_mt_ complex as described earlier^7^. Five micromolar of EF-G2_mt,_ together with 200 µM GMPPCP, was added to the 55S•RRF_mt_ complex and incubated for various timepoints (5 s, 30 s and 2 min) at 37 °C to obtain the 55S•RRF_mt_•EF-G2_mt_•GMPPCP complex, which was immediately utilized for the cryo-EM grid preparation.

### GTPase-Glo assay

GTPase activity was measured using the GTPase-Glo™ Assay by Promega and carried out as described^75^. In brief, a 10 µl reaction consisting of 0.1 µM ribosomes, 0.25 µM either bacterial (*E. coli*) EF-G or EF-G1_mt_ or EF-G2_mt_ and 1 µM GTP were incubated at room temperature in GTPase-Glo™ buffer in the absence and presence of varying amounts of fusidic acid (FA) for 1 h. Ten microliter of Reconstituted GTPase-Glo™ reagent was added to the reaction and left shaking at room temperature for 30 min. Finally, 20 µL of detection reagent was added, incubated for 10 min at room temperature and luminescence was measured in a Turner Biosystems Veritas™ Microplate Luminometer after 10 min. A negative control that contained only 1 µM GTP and a positive control that contained no FA were used each time. Luminescence was measured at FA concentrations of 1, 10, 100, and 1000 µM. Data was collected in duplicates and each duplicate dataset was normalized between 0 and 1 using the equation:

1 $${x}_{{{\rm{norm}}}}=\frac{x-{x}_{{{\rm{min}} }}}{{x}_{{{\rm{max}} }}-{x}_{{{\rm{min}} }}}.$$

As GTPase activity and luminescence measured had an inverse relationship, % GTPase activity was calculated with the formula %GTPase activity = (1 – *x*_norm_) × 100. The values at each point were then averaged and standard deviation was calculated. The raw luminescence values are presented in Table S1. The plot was generated using Python library Matplotlib.

### Cryo-electron microscopy and image processing

Home-made carbon was coated as a continuous layer (~50 Å thick) onto Quantifoil holey carbon  1.2/1.3 copper grids, which were then glow-discharged for 30 s on a plasma sterilizer. Four microliter of the sample was loaded to each of the multiple grids, incubated for 15 s at 4 °C and 100% humidity, and then blotted for 4 s before flash-freezing into the liquid ethane using a Vitrobot IV (FEI). Data was acquired on a Titan Krios electron microscope equipped with a Gatan K2 summit direct-electron detecting camera at 300 KV. A defocus range of −1.0 to −3.0 µm was used at a calibrated magnification of 22,500×, yielding a pixel size of 1.0732 Å on the object scale. A dose rate of 8.25 electrons per pixel per second and an exposure time of 10 seconds resulted in a total dose of 71.6 eÅ^−2^. CryoSPARC 3.0.1^76^ was employed for all the subsequent data processing steps. After applying full-frame motion correction to all 50 movie frames corresponding to each of the 21,752 micrographs that were collected, 142 bad micrographs were deselected after determining their contrast transfer function (CTF) using CTFFIND4^77^. From the remaining 21,610 micrographs, a total of 5,046,906 particles were autopicked, which were then subjected to local motion correction and then 4,049,952 particles were retained. This step was followed by reference-free 2D classification which allowed us to further separate the good particles (1,140,751) from the bad particles (2,909,201) based on the 2D averages. Reference-based 3D classification was employed first to separate the particles into intact 55S mitoribosomes (162,354 particles), 39S subunits (419,699 particles), 28S subunits (235,896 particles) and poorly aligned particles (322,802 particles). To obtain more homogenous sub-populations, particles corresponding to the 55S mitoribosomes, 39S and 28S subunits were each subjected to additional rounds of 3D classification that finally yielded three stable classes representing three distinct functional states formed during the human mitoribosome recycling process. Two 55S mitoribosome classes, Class I (93,212 particles) and Class II (28,929 particles), were finally refined to 3.49 and 3.91 Å, respectively, while the 39S subunit Class III (132,008 particles) was refined to 3.15 Å.

### Model building and optimization

Coordinates corresponding to the small and large subunits from our published human mitoribosome structures^6^ (PDB ID: 6VLZ) were docked independently as rigid bodies into the corresponding cryo-EM density maps of the Class I, Class II, and Class III complexes using Chimera 1.14 (Pettersen EF 2004). To obtain optimal fitting into our cryo-EM densities, the models were subsequently refined in PHENIX 1.14 (Adams PD 2010) using the “real-space refinement” function. Coordinates belonging to the human RRF_mt_ (Koripella et al.^43^) were placed independently into the corresponding cryo-EM densities of all the three maps as rigid bodies using Chimera 1.14^78^ and the models were further real-space refined in PHENIX 1.14^79^ to achieve optimal accommodation into the cryo-EM densities. The primary aa sequence of EF-G2_mt_ was submitted to the I-TASSER server^80^ to generate the initial EF-G2_mt_ homology model that was used to interpret the corresponding cryo-EM density. Segments in the homology model that do not fully accommodate into the corresponding EF-G2_mt_ density were modeled de novo using Chimera 1.14^78^ and COOT 0.9.5^81^. For the final optimization of the model into the cryo-EM density, the “Real-space refinement” function in PHENIX 1.14^79^ was utilized. Validation reports for all the models were obtained from the Molprobity server^82^ and the overall statistics of EM reconstruction and molecular modeling are listed in Table S2. All the figures in the manuscript were generated using ChimeraX 1.1.1^83^.

### Reporting summary

Further information on research design is available in the Nature Research Reporting Summary linked to this article.

## Supplementary information

Supplementary Information

Reporting Summary

## Data Availability

The cryo-EM density maps and the atomic coordinates have been deposited in the Electron Microscopy Data Bank and the Protein Data Bank under the accession codes EMD-23096 and PDB ID 7L08 for the RRF_mt_-bound human 55S mitoribosome (Complex I), EMD-23114 for the RRF_mt_•EF-G2_mt_-bound human 55S mitoribosome (Complex II), EMD-23121 [https://www.ebi.ac.uk/pdbe/entry/emdb/EMD-23121] and PDB ID 7L20 for the RRF_mt_•EF-G2_mt_-bound human 39S mitoribosome (Complex III). All the raw image stacks used in the 3D reconstructions are being made available through EMPIAR [https://www.ebi.ac.uk/pdbe/emdb/empiar/entry/10703].
