# Supplementary material for: Distinct mechanisms of the human mitoribosome recycling and antibiotic resistance
Source: Nat Commun. 2021 Jun 14;12:3607. doi: 10.1038/s41467-021-23726-4 (PMC8203779; doi:10.1038/s41467-021-23726-4)
Supplement: Supplementary file 1 — Supplementary Information [file 41467_2021_23726_MOESM1_ESM.pdf]

Supplementary Information

Structures of the human mitochondrial ribosome recycling complexes reveal distinct mechanisms of recycling and antibiotic resistance

Ravi Kiran Koripella[1], Ayush Deep[1], Ekansh K. Agrawal[1], Pooja Keshavan[1], Nilesh K. Banavali[1,3], and Rajendra K. Agrawal[1,3,*]

[1]Wadsworth Center, New York State Department of Health, Empire State Plaza, Albany, New York 12201; and

[3]Department of Biomedical Sciences, University at Albany, SUNY, New York 12201-0509.

*Corresponding author: rajendra.agrawal@health.ny.gov

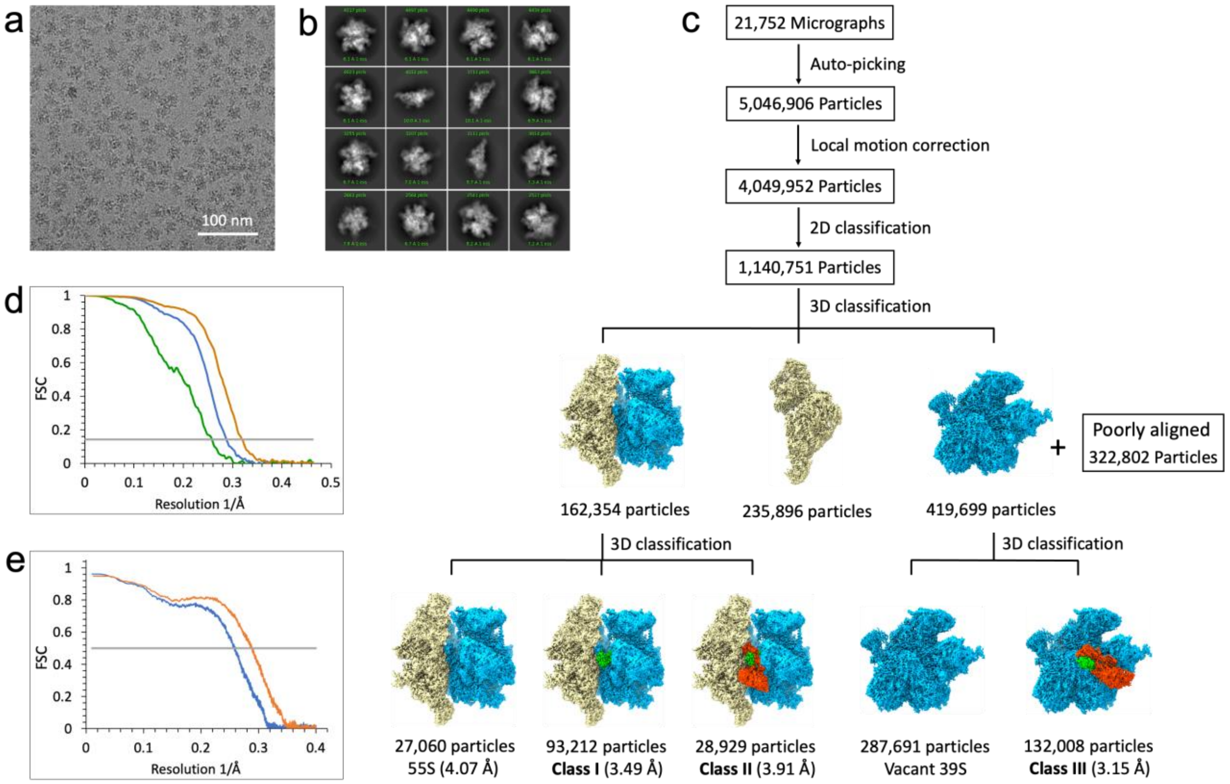

Supplementary Figure 1. Image processing of the human mitochondrial ribosome recycling complexes. (**a**) A representative electron micrograph obtained for the human mitoribosomal 55S•RRF$_{mt}$•EF-G2$_{mt}$•GMPPCP complex. (**b**) Representative two-dimensional (2D) class averages used in initial three-dimensional (3D) reconstructions. (**c**) Flow-chart showing results of 3D classifications and refinements. The selected 2D averages (1,140,751 particles) were subjected to several rounds of reference-based 3D classification to separate the intact 55S mitoribosomes (162,354 particles) from the 28S subunits (235,896 particles), the 39S subunits (419,699 particles) and the poorly aligned images (322,802 particles). Further classification of the 55S mitoribosomes yielded three stable classes. The 55S maps that contained bound ligands were refined to 3.49 Å (Class I) and 3.91 Å (Class II). Particles corresponding to the 39S subunit were subjected to additional rounds of 3D classification that finally yielded a stable 39S class (Class III) bound with both factors, RRF$_{mt}$ and EF-G2$_{mt}$. This 39S class was refined to 3.15 Å. **(d)** Fourier-shell correlation (FSC) plots of the Classs I (blue), Class II (green) and Class III (orange) complexes. **(e)** Map vs model FSC plots of the Class I (blue) and Class III (orange) complexes.

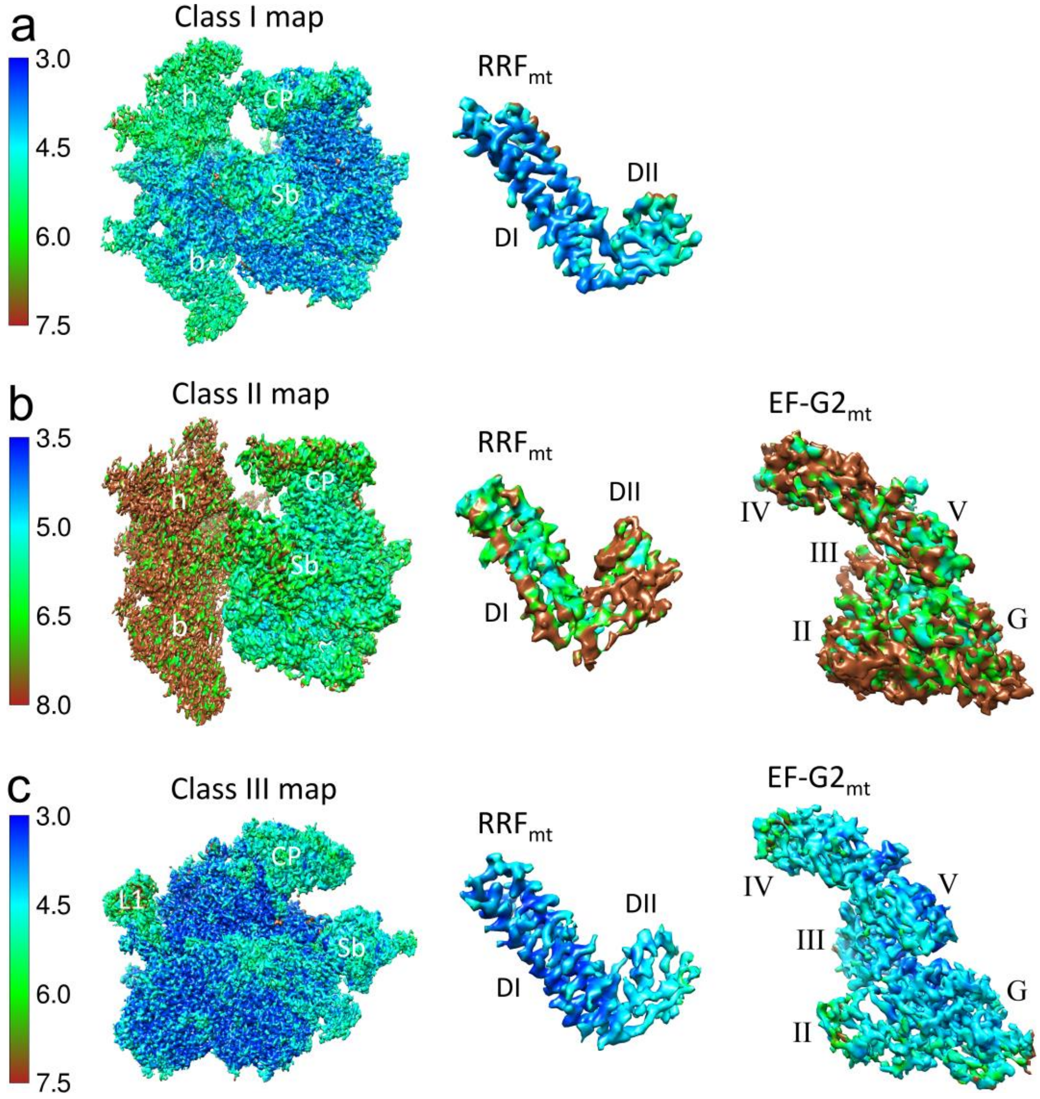

Supplementary Figure 2. Local surface resolution of the human mitoribosome recycling complexes. (a-c) The Left panels show the local resolution of Class I, Class II and Class III maps, respectively. The middle panels and the right panels show the local resolution of RRF_{mt} and $EF\text{-}G_{mt}$ components extracted from the corresponding cryo-EM maps displayed in the left panels.

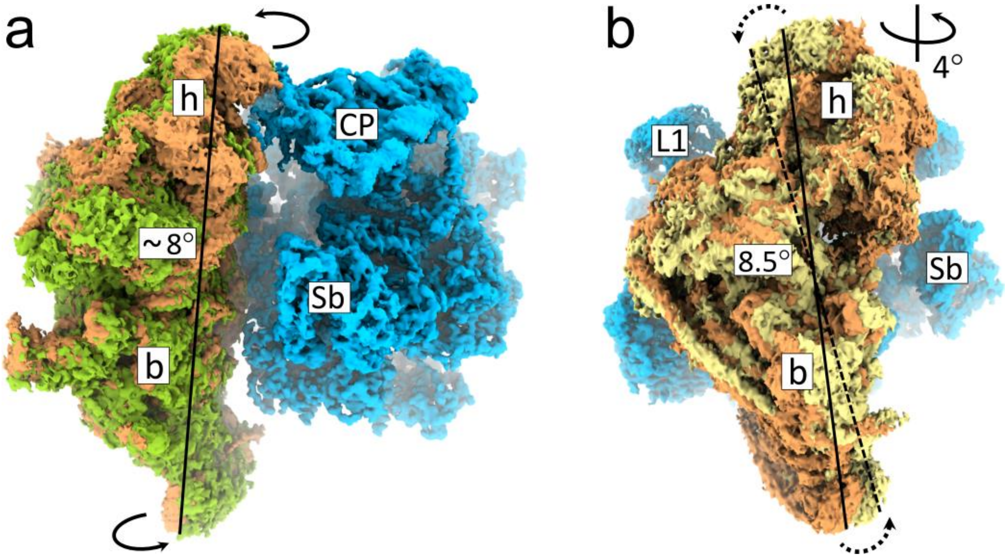

Supplementary Figure 3. Conformational states of the 28S subunit in the Class I and Class II mitoribosomal recycling complexes. (**a**) Comparison of our factor-free 55S cryo-EM map with the published factor-free human 55S mitoribosome [1] (light brown) showed that 28S subunit (light green) was rotated by ~8° around its long axis with its shoulder side moving closer to the large subunit while its platform side moving away from it. **(b)** Superimposition of the Class I cryo-EM map with the factor-free 55S mitoribosome (light brown) [1] revealed an overall ~8.5° rotation of the 28S subunit (yellow) in an anti-clockwise direction relative to the 39S subunit (blue). Additionally, the head domain of the 28S subunit rotated by ~4° towards the tRNA exit (E) site in a roughly orthogonal direction to the inter-subunit motion. In both the panels, landmarks of the 28S subunit: h, head; b, body. Landmarks of the 39S subunit: CP, central protuberance; Sb, stalk base; L1, MRP uL1m.

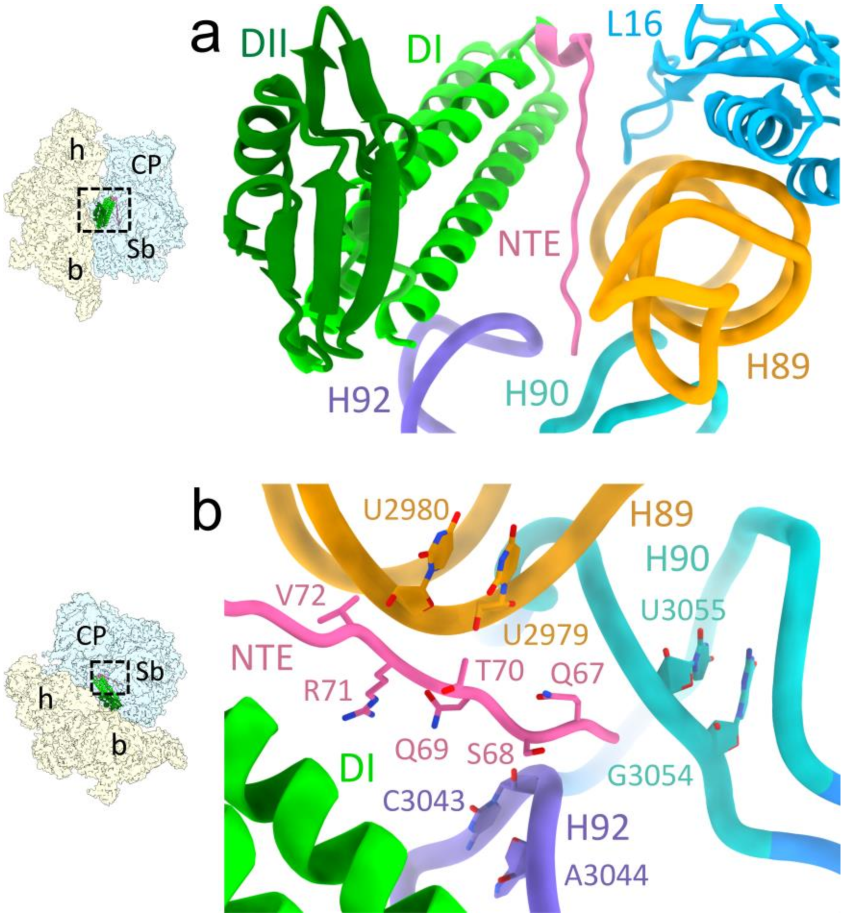

Supplementary Figure 4. Interactions of RRF$_{mt}$ NTE with the mitoribosomal components. (a) Simultaneous interactions of RRF$_{mt}$ NTE (pink) of RRF$_{mt}$ with various functionally important helices of the 16S rRNA such as H89 (orange), H90 (turquoise), and H92 (purple), and MRP uL16m (light blue). **(b)** Magnified view showing that the mitoribosomal components involved in these interactions remain essentially the same as described earlier (Koripella et al., 2019), despite an altered conformation of NTE. Thumbnail to the left depicts an overall orientation of the 55S mitoribosome, with semitransparent 28S (yellow) and 39S (blue) subunits, and overlaid positions of ligands. Landmarks on the thumbnail: h, head, and b, body of the 28S subunit, and CP, central protuberance; Sb, stalk base of the 39S subunit.

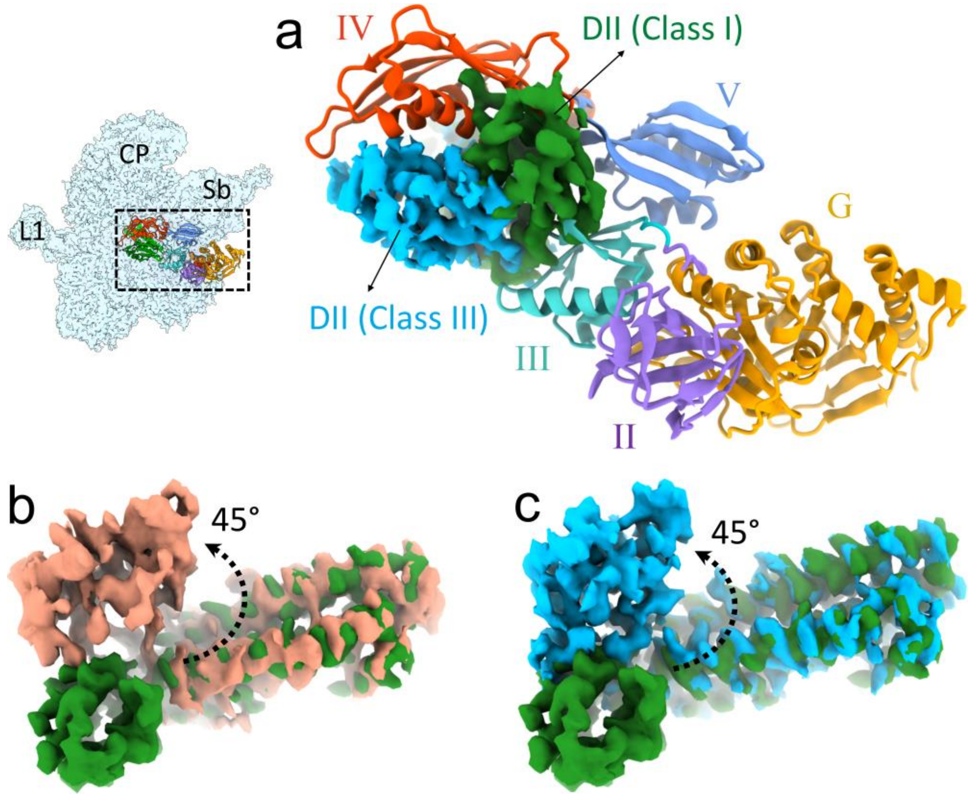

Supplementary Figure 5. Domain II of RRF_{mt} undergoes large conformational change to avoid steric clash with EF-$G2_{mt}$. (a) Superposition of the 55S•RRF_{mt} complex (Class I) with the 39S•RRF_{mt}•EF-$G2_{mt}$•GMPPCP complex (Class III) shows that the orientation of domain II (green) in Class I complex would prevent the binding of EF-$G2_{mt}$ to the RRF_{mt}-bound 55S mitoribosome due to direct steric conflict with the EF-$G2_{mt}$'s domains III (cyan), IV (red) and V (blue). **(b, c)** comparison of orientations of RRF_{mt} domain II in Class I (green) Class II (light brown) and Class III (light blue) complexes. Thumbnail to the top left depicts an overall orientation of the 39S subunit (semitransparent blue) with overlaid positions of the ligands for panel A. Landmarks on the thumbnail: CP, central protuberance; Sb, stalk base; L1, MRP uL1m.

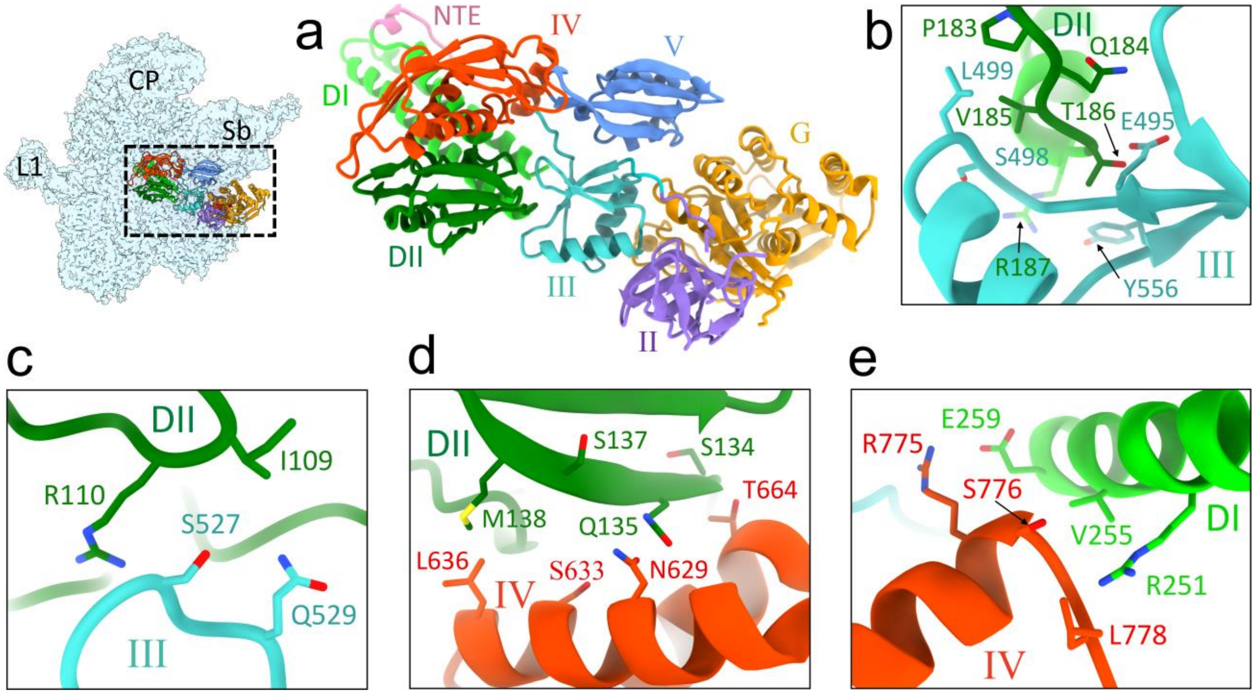

Supplementary Figure 6. Interactions between RRF$_{mt}$ and EF-G2$_{mt}$ in the Class III complex. **(a)** Domain II of RRF$_{mt}$ (dark green) is positioned in the pocket formed between domains III (cyan), IV (red) and V (blue) of EF-G2$_{mt}$. Thumbnail to the left of panel A depicts an overall orientation of the 39S subunit (semitransparent blue) with overlaid positions of the ligands. Landmarks on the thumbnail: CP, central protuberance; Sb, stalk base; L1, MRP uL1m. **(b, c)** Interactions of RRF$_{mt}$ domain II with domain III of EF-G2$_{mt}$. **(d)** Interactions of RRF$_{mt}$ domain II with domain IV of EF-G2$_{mt}$. **(e)** Interactions of RRF$_{mt}$ domain I with domain IV of EF-G2$_{mt}$.

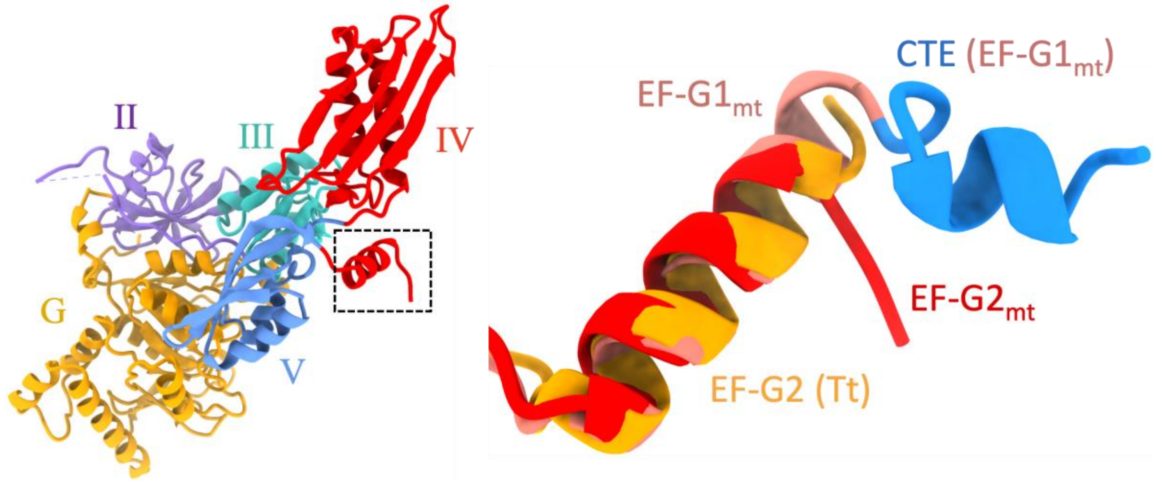

Supplementary Figure 7. Comparison of the domain IV C-terminal α-helical regions among the EF-G variants. In EF-$G1_{mt}$, the domain IV C-terminal α-helix (salmon) along with its CTE (blue) is much larger compared to EF-$G2_{mt}$ (red) and *T. thermophilus* EF-G2[2] (orange).

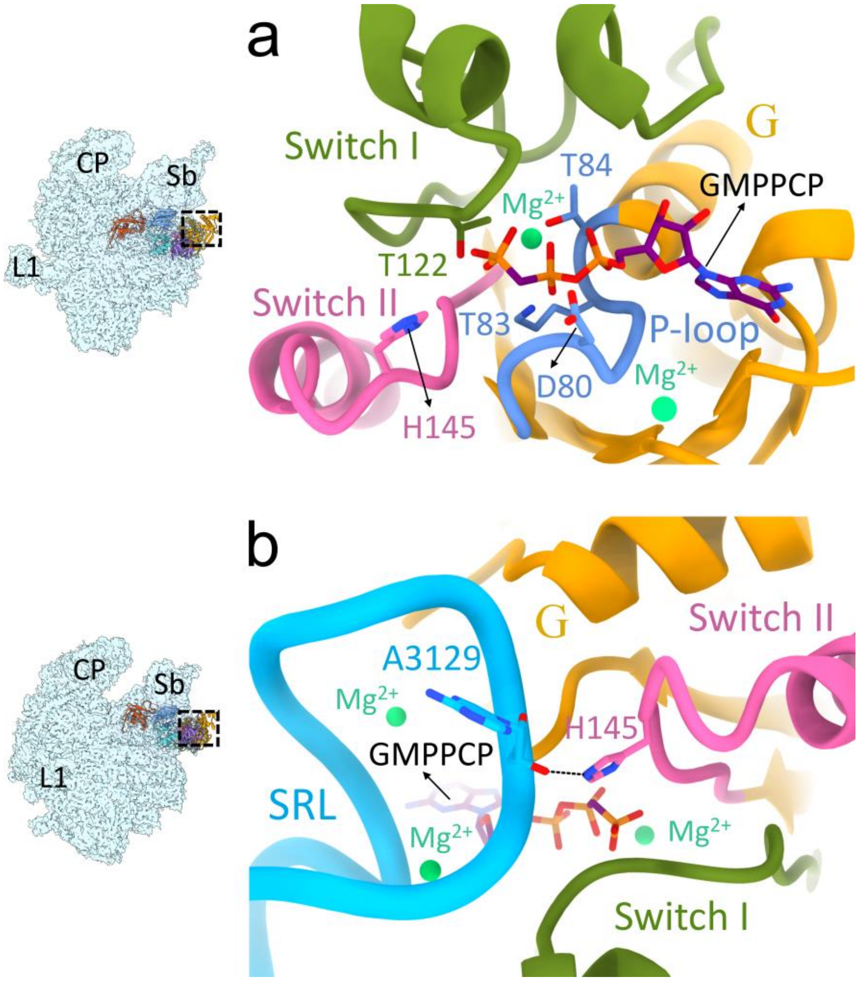

Supplementary Figure 8. Interactions of the functionally essential elements within the G domain of EF-G2$_{mt}$ with GMPPCP and the SRL. (**a**) GMPPCP is stably held in the nucleotide binding pocket through multiple interactions with conserved aa residues of the functionally important elements of the G domain such as Switch I (green), Switch II (pink) and the P-loop (blue). Magnesium ions present in the vicinity are shown as light green spheres. **(b)** The conserved H145 residue that is known to play a central role during GTP hydrolysis [3] is stabilized in its active conformation by interacting with the sugar moiety of the highly conserved A3129 residue from the SRL. Colors of the G domain components in the panels **a** and **b** are matched. Thumbnails to the left depict overall orientations of the 39S subunit (semitransparent blue) and overlaid ligands. Landmarks on the thumbnail: CP, central protuberance; Sb, stalk base; L1, MRP uL1m.

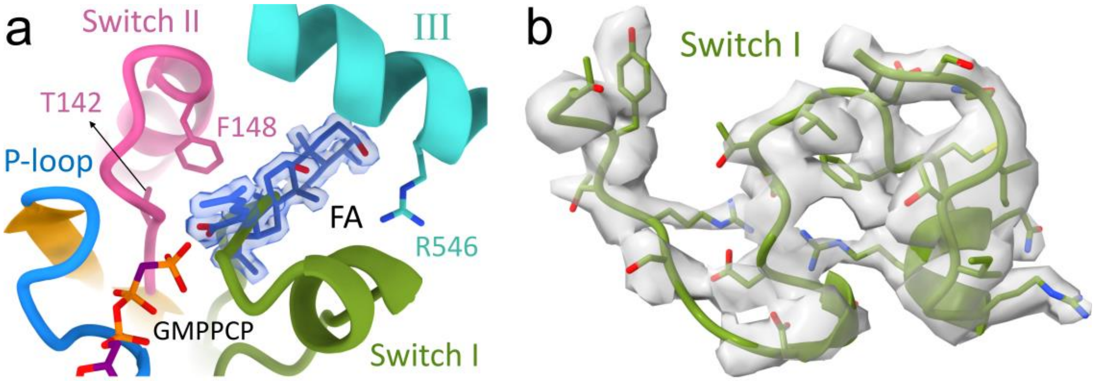

c

```
EF-G:       TAAVTTCFWKDHRINIIDTPGHVDFTIEVERSMRVLDGAIVV   107
EF-G1mt:    QSAATYTMWKDVNINIIDTPGHVDFTIEVERALRVLDGAVLV   144
EF-G2mt:    QSAAVTFDWKGYRVNLIDTPGHVDFTLEVERCLRVLDGAVAV   165

EF-G:       THPETGQTIISGMGELHLEIIVDRLKREFKVDANVGKPQVAY   483
EF-G1mt:    FDTENKETVISGMGELHLEIYAQRLEREYGCPCITGKPKVAF   525
EF-G2mt:    LDPDSGQTVLCGMGELHIEIIHDRIKREYGLETYLGPLQVAY   564
```

Supplementary Figure 9. Stabilized Switch I region in EF-G2$_{mt}$ prevents FA from accessing its binding site. (a) Superimposition of the FA molecule (dark blue) from the bacterial 70S•EF-G•GDP•FA complex [4] into the G domain of EF-G2$_{mt}$ in our 39S•RRF$_{mt}$•EF-G2$_{mt}$•GMPPCP complex reveals that the putative FA binding site is sterically blocked by the switch I region. Key aa residues known to be important for the stable binding of FA are shown with sidechains. Colors of the G domain components are similar as in supplementary figure 7. **(b)** Cryo-EM density corresponding to the switch I region (green) extracted from the 39S•RRF$_{mt}$•EF-G2$_{mt}$•GMPPCP complex. **(c)** The aa residues that are known to be necessary for the stable binding of FA [4] are conserved in all the three EF-Gs and are highlighted in pink.

Supplementary Table 1. Raw luminescence values obtained for GTpase activities of **(a)** *E. coli* EF-G **(b)** EF-G1$_{mt}$, and **(c)** EF-G2$_{mt}$, using the GTPase-Glo™ Assay.

a

E. coli EF-G + *E. coli* 70S						
	Negative control; GTP only	Positive control; GTP + GTPase + ribosomes	Positive control + 1 uM FA	Positive control + 10 uM FA	Positive control + 100 uM FA	Positive control + 1000 uM FA
Replicate 1	430,411	87,574	52,556	121,740	293,532	31,7535
Replicate 2	396,292	58,556	62,585	274,961	329,175	413,717
Average	413,351.5	73,065	57,570.5	198,350.5	311,353.5	365,626
Normalized	1	0.04355	0	0.39569	0.71331	0.86585
% GTPase activity	0	95.645	100	60.431	28.669	13.414

b

H. sapiens EF-G1mt + *H. sapiens* 55S						
	Negative control; GTP only	Positive control; GTP + GTPase + ribosomes	Positive control + 1 uM FA	Positive control + 10 uM FA	Positive control + 100 uM FA	Positive control + 1000 uM FA
Replicate 1	263,604	90,747	63,577	77,195	69,957	65,802
Replicate 2	345,884	127,155	87,979	73,719	118,776	70,889
Average	304,744	108,951	75,778	75,457	94,366.5	68,345.5
Normalized	1	0.17177	0.03144	0.03008	0.11007	0
% GTPase activity	0	82.823	96.856	96.991	88.993	100

c

H. sapiens EF-G2mt + *H. sapiens* 55S						
	Negative control; GTP only	Positive control; GTP + GTPase + ribosomes	Positive control + 1 uM FA	Positive control + 10 uM FA	Positive control + 100 uM FA	Positive control + 1000 uM FA
Replicate 1	655,837	115,842	95,497	81,520	106,816	131,740
Replicate 2	580,399	127,991	101,928	119,485	125,681	148,052
Average	618,118	121,916.5	98,712.5	100,502.5	116,248.5	139,896
Normalized	1	0.04467	0	0.00345	0.03376	0.07929
% GTPase activity	0	95.533	100	99.655	96.624	92.071

Supplementary Table 2. Data collection, Refinement and Model validation.

Description	55S•RRF_{mt} (Class I)	55S•RRF_{mt}•EF-$G2_{mt}$ (Class III)
**Data collection and Refinement**		
Microscope	FEI Titan Krios	
Voltage (kV)	300	
Pixel size (Å)	1.073	
Defocus range (µm)	1.0 to 3.0	
Average e^- dose per image (e^-/Å^2)	71.6	
Software	cryoSPARC	
Particles (initial)	1,140,751	
Particles (final)	93,212	132,008
Symmetry	C1	C1
FSC-threshold	0.143	0.143
Resolution (Å)	3.49	3.15
Map-sharpening *B* factor (Å^2) overall	51.4	55.6
**RMS deviations**		
Bonds (Å)	0.00	0.01
Angles (°)	0.05	0.05
**Molprobity clashscore**	1.97 (77nd)	2.03 (74th)
Clashscore, all atoms	10.33	10.97
**Rotamer outliers (%)**	0.68	0.84
**Ramachandran plot**		
Favored (%)	93.24	92.23
Outliers (%)	0.19	0.50
**RNA**		
Correct sugar puckers (%)	98.42	98.17
Angle outliers (%)	0.01	0.01
Bond outliers (%)	0.00	0.00
Good backbone conformations (%)	76.24	77.12
**Model composition**		
RNA bases<br>Protein residues	2,527<br>14,369	1,583<br>9,322
**Accession codes**		
Cryo-EM maps	EMD-23096	EMD-23121
PDB ID	7L08	7L20

Supplementary References

1. Amunts, A., Brown, A., Toots, J., Scheres, S.H.W. & Ramakrishnan, V. Ribosome. The structure of the human mitochondrial ribosome. *Science* **348**, 95-98 (2015).
2. Connell, S.R. et al. Structural basis for interaction of the ribosome with the switch regions of GTP-bound elongation factors. *Mol Cell* **25**, 751-64 (2007).
3. Tourigny, D.S., Fernandez, I.S., Kelley, A.C. & Ramakrishnan, V. Elongation factor G bound to the ribosome in an intermediate state of translocation. *Science* **340**, 1235490 (2013).
4. Gao, Y.G. et al. The structure of the ribosome with elongation factor G trapped in the posttranslocational state. *Science* **326**, 694-9 (2009).
